# Plasma Proteomics to Identify Drug Targets for Ischemic Heart Disease

**DOI:** 10.1016/j.jacc.2023.09.804

**Published:** 2023-11-14

**Authors:** Mohsen Mazidi, Neil Wright, Pang Yao, Christiana Kartsonaki, Iona Y. Millwood, Hannah Fry, Saredo Said, Alfred Pozarickij, Pei Pei, Yiping Chen, Daniel Avery, Huaidong Du, Dan Valle Schmidt, Ling Yang, Jun Lv, Canqing Yu, Junshi Chen, Michael Hill, Michael V. Holmes, Joanna M.M. Howson, Richard Peto, Rory Collins, Derrick A. Bennett, Robin G. Walters, Liming Li, Robert Clarke, Zhengming Chen, China Kadoorie Biobank Collaborative Group, Junshi Chen, Zhengming Chen, Robert Clarke, Rory Collins, Liming Li, Chen Wang, Jun Lv, Richard Peto, Robin Walters, Daniel Avery, Maxim Barnard, Derrick Bennett, Ruth Boxall, Sushila Burgess, Ka Hung Chan, Yiping Chen, Zhengming Chen, Johnathan Clarke, Robert Clarke, Huaidong Du, Ahmed Edris Mohamed, Hannah Fry, Simon Gilbert, Pek Kei Im, Andri Iona, Maria Kakkoura, Christiana Kartsonaki, Hubert Lam, Kuang Lin, James Liu, Mohsen Mazidi, Iona Millwood, Sam Morris, Qunhua Nie, Alfred Pozarickij, Paul Ryder, Saredo Said, Dan Schmidt, Becky Stevens, Iain Turnbull, Robin Walters, Baihan Wang, Lin Wang, Neil Wright, Ling Yang, Xiaoming Yang, Pang Yao, Xiao Han, Can Hou, Qingmei Xia, Chao Liu, Jun Lv, Pei, Dianjanyi Sun, Canqing Yu, Naying Chen, Duo Liu, Zhenzhu Tang, Ningyu Chen, Qilian Jiang, Jian Lan, Mingqiang Li, Yun Liu, Fanwen Meng, Jinhuai Meng, Rong Pan, Yulu Qin, Ping Wang, Sisi Wang, Liuping Wei, Liyuan Zhou, Caixia Dong, Pengfei Ge, Xiaolan Ren, Zhongxiao Li, Enke Mao, Tao Wang, Hui Zhang, Xi Zhang, Jinyan Chen, Ximin Hu, Xiaohuan Wang, Zhendong Guo, Huimei Li, Yilei Li, Min Weng, Shukuan Wu, Shichun Yan, Mingyuan Zou, Xue Zhou, Ziyan Guo, Quan Kang, Yanjie Li, Bo Yu, Qinai Xu, Liang Chang, Lei Fan, Shixian Feng, Ding Zhang, Gang Zhou, Yulian Gao, Tianyou He, Pan He, Chen Hu, Huarong Sun, Xukui Zhang, Biyun Chen, Zhongxi Fu, Yuelong Huang, Huilin Liu, Qiaohua Xu, Li Yin, Huajun Long, Xin Xu, Hao Zhang, Libo Zhang, Jian Su, Ran Tao, Ming Wu, Jie Yang, Jinyi Zhou, Yonglin Zhou, Yihe Hu, Yujie Hua, Jianrong Jin, Fang Liu, Jingchao Liu, Yan Lu, Liangcai Ma, Aiyu Tang, Jun Zhang, Liang Cheng, Ranran Du, Ruqin Gao, Feifei Li, Shanpeng Li, Yongmei Liu, Feng Ning, Zengchang Pang, Xiaohui Sun, Xiaocao Tian, Shaojie Wang, Yaoming Zhai, Hua Zhang, Wei Hou, Silu Lv, Junzheng Wang, Xiaofang Chen, Xianping Wu, Ningmei Zhang, Weiwei Zhou, Xiaofang Chen, Jianguo Li, Jiaqiu Liu, Guojin Luo, Qiang Sun, Xunfu Zhong, Weiwei Gong, Ruying Hu, Hao Wang, Meng Wang, Min Yu, Lingli Chen, Qijun Gu, Dongxia Pan, Chunmei Wang, Kaixu Xie, Xiaoyi Zhang

**Affiliations:** a Clinical Trial Service Unit, Nuffield Department of Population Health, University of Oxford, Oxford, United Kingdom; b Medical Research Council Health Research Unit, Nuffield Department of Population Health, University of Oxford, Oxford, United Kingdom; c Peking University Center for Public Health and Epidemic Preparedness and Response, Beijing, China; d Department of Epidemiology and Biostatistics, School of Public Health, Peking University Health Science Center, Beijing, China; e Key Laboratory of Epidemiology of Major (Peking University), Ministry of Education, Beijing, China; f China National Center for Food Risk Assessment, Beijing, China; g Novo Nordisk Research Centre, Oxford, United Kingdom

**Keywords:** diverse populations, drug target, genetics, ischemic heart disease, Mendelian randomization, plasma proteomics, prospective studies

## Abstract

**Background:**

Integrated analyses of plasma proteomic and genetic markers in prospective studies can clarify the causal relevance of proteins and discover novel targets for ischemic heart disease (IHD) and other diseases.

**Objectives:**

The purpose of this study was to examine associations of proteomics and genetics data with IHD in population studies to discover novel preventive treatments.

**Methods:**

We conducted a nested case-cohort study in the China Kadoorie Biobank (CKB) involving 1,971 incident IHD cases and 2,001 subcohort participants who were genotyped and free of prior cardiovascular disease. We measured 1,463 proteins in the stored baseline samples using the OLINK EXPLORE panel. Cox regression yielded adjusted HRs for IHD associated with individual proteins after accounting for multiple testing. Moreover, *cis*-protein quantitative loci (pQTLs) identified for proteins in genome-wide association studies of CKB and of UK Biobank were used as instrumental variables in separate 2-sample Mendelian randomization (MR) studies involving global CARDIOGRAM+C4D consortium (210,842 IHD cases and 1,378,170 controls).

**Results:**

Overall 361 proteins were significantly associated at false discovery rate <0.05 with risk of IHD (349 positively, 12 inversely) in CKB, including N-terminal prohormone of brain natriuretic peptide and proprotein convertase subtilisin/kexin type 9. Of these 361 proteins, 212 had *cis*-pQTLs in CKB, and MR analyses of 198 variants in CARDIOGRAM+C4D identified 13 proteins that showed potentially causal associations with IHD. Independent MR analyses of 307 *cis*-pQTLs identified in Europeans replicated associations for 4 proteins (FURIN, proteinase-activated receptor-1, Asialoglycoprotein receptor-1, and matrix metalloproteinase-3). Further downstream analyses showed that FURIN, which is highly expressed in endothelial cells, is a potential novel target and matrix metalloproteinase-3 a potential repurposing target for IHD.

**Conclusions:**

Integrated analyses of proteomic and genetic data in Chinese and European adults provided causal support for FURIN and multiple other proteins as potential novel drug targets for treatment of IHD.

## Central Illustration

**Figure:**
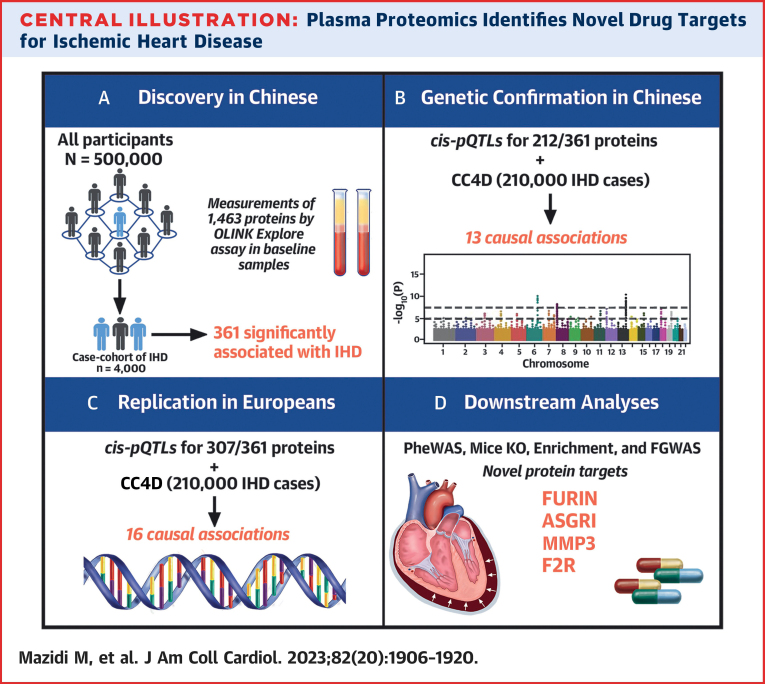

Ischemic heart disease (IHD) accounts for about 9 million annual deaths worldwide, and the incidence rates are increasing in many populations, including China.^1^ Tobacco smoking, hypertension, dyslipidemia, and adiposity are established risk factors for IHD, but other risk factors remain to be discovered. Recently, genome-wide association studies (GWAS) of IHD in primarily European ancestry populations have identified >240 common genetic variants,^2^^,^^3^ but the mechanisms underlying many of these associations remain to be elucidated.^2^ Most drug targets are enzymes, transport, or structural proteins, and novel assays that measure plasma levels of large numbers of proteins, particularly when integrated with genetic data, could discover novel risk factors and potential therapeutic targets for IHD and improve risk prediction and early detection of IHD.^4^

Previous studies of plasma proteins and IHD have highlighted the roles of apolipoproteins,^5^ inflammatory proteins (eg, interleukin [IL]-6),^6^ or early markers of disease (troponins or N-terminal prohormone of brain natriuretic peptide [NT-proBNP]).^7^ Advances in proteomic assays now enable measurement of thousands of proteins in blood,^8^ and their application in prospective studies have identified novel biomarkers for IHD.^9^ However, previous proteomics studies of IHD have been confined to European populations,^10^ and many lacked concomitant genetic data to assess and clarify the causal relevance of any observed associations,^4^^,^^9^^,^^11^ greatly limiting the translational potential of such study findings.

We present integrated analyses of observational and genetic data for 1,463 proteins with risk of incident IHD in a nested case-cohort study within the prospective China Kadoorie Biobank (CKB). The study aimed to: 1) identify proteins associated with risk of IHD independent of established cardiovascular disease (CVD) risk factors; 2) use *cis*-protein quantitative loci (pQTLs) identified in GWAS of CKB and UK Biobank (UKB) for these proteins to assess their causal relevance for IHD in 2-sample Mendelian randomization (MR) analyses in a global IHD GWAS consortium; and 3) explore mechanisms linking such proteins with IHD using enrichment analyses, tissue expression, and single-gene knockout (KO) models.

## Methods

### Study population and design

Details of the study design, methods, and participants in CKB have been previously reported.^12^ In brief, CKB recruited >512,000 adults aged 30 to 79 years from 10 diverse (5 urban, 5 rural) regions in China during 2004-2008. The baseline survey included a laptop-based questionnaire covering sociodemographic factors, lifestyle, medical history and medication use, and physical measurements (eg, height, weight, waist circumference, blood pressure, and lung function). Study participants provided a 10-mL nonfasting blood sample (with time since last meal recorded) for long-term storage in liquid nitrogen. Prior international, national, and local ethical approvals for the study were obtained and all participants provided written informed consent. After enrollment, the vital status and occurrence of specific diseases was monitored based on linkage to death and disease registers and to the National Health Insurance system for all hospital admissions.^12^ All causes of death or incident diseases were coded using ICD-10 codes by trained health workers, blinded to baseline information and integrated centrally.

The CKB complies with all required ethical standards for medical research on human subjects. Ethical approvals were obtained and have been maintained by the relevant institutional ethical research committees in the United Kingdom and China. All participants provided written informed consent.

**Figure 1:**
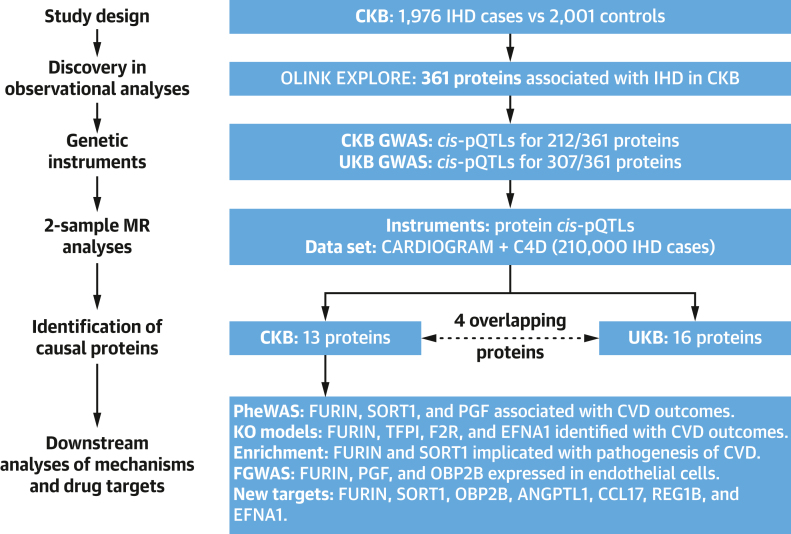
Summary of Study Design, Analytic Approaches, and Key Findings The study design involved observational and genetic analyses in the China Kadoorie Biobank (CKB) and UK Biobank (UKB) studies. The observational associations of proteomics with ischemic heart disease (IHD) were replicated using Mendelian randomization (MR) approaches using instrumental variables discovered in CKB and replicated in UKB. The downstream analyses included phenome-wide associations (PheWAS), knockout models (KO), assessment of enrichment, and functional genome-wide association study analyses (FGWAS), respectively.

The present study involved a case-cohort design (Figure 1, Central Illustration) comprising 1,937 incident cases of IHD (ICD-10 codes: I20, I22-I25) that were accrued during a 12-year follow-up before January 1, 2019 and a subcohort of 2,001 participants. The IHD cases were a random sample of incident IHD cases that had GWAS data and no prior history of CVD, and excluded statin users at enrollment. The subcohort participants were randomly selected from a population subset of 69,353 genotyped participants, but were genetically unrelated to each other and had no prior history of CVD or statin use at baseline.

**Central Illustration:**
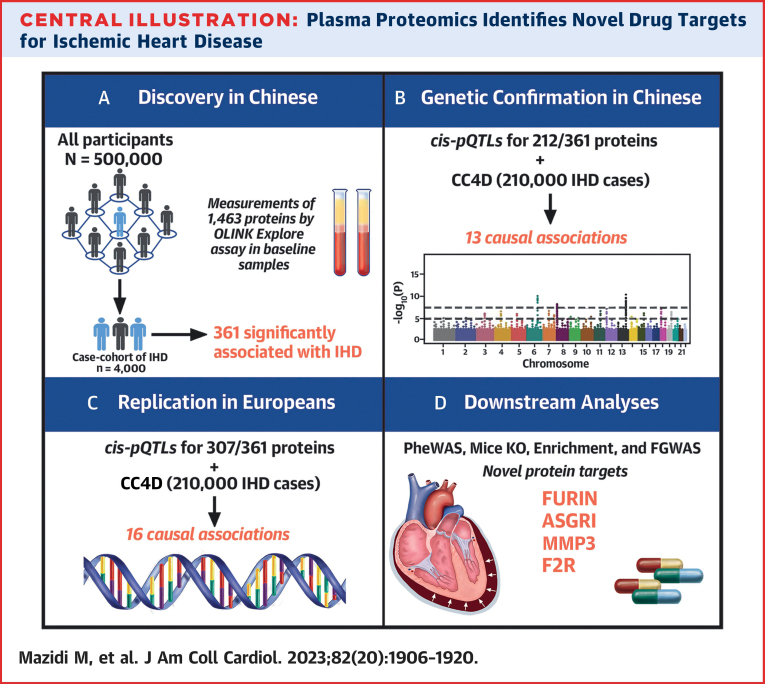
Plasma Proteomics Identifies Novel Drug Targets for Ischemic Heart Disease The study involved: (A) analyses of associations of 1,463 proteins with ischemic heart disease (IHD) in Chinese adults; (B) confirmation of associations of cis- protein quantitative loci (pQTLs) for such associated proteins using genetic analyses in CARDIOGRAM+C4D (CC4D); (C) replication of cis-pQTLs in Europeans; and (D) downstream analyses to discover novel drug targets for treatment of IHD. ASGR1 = asialoglycoprotein receptor 1; F2R = proteinase-activated receptor 1; FGWAS = functional genome-wide association study analyses; IHD = ischemic heart disease; KO = knockout; MMP3 = matrix metalloproteinase-3; PheWAS = phenome-wide associations.

### Proteomics assays

Plasma levels of 1,463 proteins were measured using the OLINK EXPLORE platform in OLINK (Uppsala, Sweden), which included 4 similar-sized panels (ie, cardiometabolic, inflammation, neurology, and oncology).^13^ Details of the proteomics assays are provided in the Supplemental Methods, Supplemental Tables 1 and 2, and Supplemental Figure 1.

### Statistical methods

For observational analyses of proteins with IHD, Cox regression models were used to estimate adjusted HRs (and 95% CIs) for IHD using the Prentice pseudo-partial likelihood for case-cohort designs.^14^ All analyses were stratified based on sex and study area, and adjusted sequentially in different models for additional CVD risk factors (Supplemental Methods). For genetic analyses involving 1- and 2-sample MR analyses, we used *cis*-pQTLs derived from GWAS of protein levels in Chinese (CKB) and European (UKB) adults to assess the causal relevance of proteins identified in CKB observational analyses.^15^

For proteins with *cis*-pQTLs identified in CKB, we undertook 1-sample MR analyses in the case-cohort sample (n = 1,937 IHD cases) and in the overall CKB genotyping data set (n = 6,499 IHD cases).^16^ We further conducted 2-sample MR analyses of the proteins identified in CKB observational analyses using: 1) *cis*-pQTLs obtained from CKB, with lookups in the CARDIOGRAM+C4D (CC4D) Consortium of European and Japanese adults that included 210,842 IHD cases and 1,378,170 controls,^2^ and also separately in Biobank Japan^17^; and 2) *cis-*pQTLs from UKB^15^ with lookups in the CC4D of European adults only (n = 181,522 cases).^2^

To explore genetic associations of *cis*-pQTLs with CVD traits, we searched PhenoScanner, which included GWAS results for >100 traits or disease outcomes,^18^ using a *P* value threshold of <10 × 5^-8^. Additional downstream analyses were also undertaken of any proteins causally associated with IHD in genetic analyses, including: 1) KO models; 2) enrichment analyses using the DAVID (Database for Annotation, Visualisation and Integrated Discovery) bioinformatics resource 6.8;^19^ 3) enrichment in chromatin in human umbilical endothelial cells tissues using functional GWAS;^20^ 4) gene expression in aortic tissues by GTEx (Genotype Tissue Expression) database (version 8); 5) details of drug target development for clinical (Tclin) or chemical (Tchem) outcomes;^21^ and 6) information on drug targets including evaluation in phase II-IV trials using DrugBank and OpenTargets databases. All the analyses were corrected for multiple testing, using FDR (false discovery rate) <0.05,^22^ or more stringent Bonferroni-corrected threshold (*P* = 0.05/1,463) as sensitivity analyses for observational associations.^23^ All analyses used R version 4.1.2.

## Results

Overall, patients with IHD were older (mean baseline age: 63.8 ± 9.2 years vs 51.2 ± 10.3 years) and more likely to be men (62.1% vs 37.9%), to be less well-educated, and to smoke than subcohort participants. IHD cases also had a higher prevalence of hypertension and diabetes and higher mean levels of systolic blood pressure (SBP), adiposity, and random blood glucose (Table 1). The median time from enrollment to diagnosis of IHD cases was 6.8 years (IQR: 4.5 years). Figure 1 provides an overview of the main analytic approaches and key findings, including observational, genetic, and relevant additional downstream analyses.

**Table 1:** Baseline Characteristics of IHD Cases and Subcohort Participants in CKB

	IHD Cases (n = 1937)	Subcohort (n = 2026)
Demographic and socioeconomic factors		
Age, y	63.8 ± 9.2	51.2 ± 10.3
Female	37.9	62.1
Urban resident	46.0	50.6
≥6 y education	51.9	52.1
Household income ≥35,000 yuan/y	15.7	19.2
Medical history and medication^a^		
Hypertension	20.1	9.8
Diabetes	8.7	3.1
Blood pressure–lowering medication	34.9	32.6
Medication for diabetes	82.0	75.9
Lifestyle factors		
Physical activity, MET-h	19.0 ± 10.4	21.3 ± 14.4
Ever-regular smoker		
Male	82.8	74.9
Female	6.5	3.3
Regular alcohol drinker		
Male	38.3	44.5
Female	5.8	2.9
Clinical measurements		
SBP, mm Hg	138.9 ± 24.5	130.5 ± 21.4
DBP, mm Hg	81.7 ± 12.7	78.0 ± 11.0
BMI, kg/m^2^	24.3 ± 3.8	23.8 ± 3.4
WC, cm	82.4 ± 10.6	80.2 ± 9.9
RBG, mmol/L	6.8 ± 3.9	6.1 ± 2.2
Fasting time, h	5.2 ± 4.4	5.0 ± 5.0

Values are mean ± SD or %. All values are adjusted for age, sex, and region, where appropriate.

BMI = body mass index; DBP = diastolic blood pressure; MET = metabolic equivalent of task h/d; RBG = random blood glucose; SBP = systolic blood pressure; WC = waist circumference.

a Medical history was based on self-report of physician-diagnosed conditions, with use of medication restricted to those reporting relevant conditions.

### Observational associations of proteins with conventional CVD risk factors and with IHD

A total of 1,009 proteins were significantly associated (at FDR <0.05) with body mass index, 928 with SBP, 833 with diabetes, and 329 with smoking, and 169 proteins with all of these risk factors (Supplemental Figure 2). In Model 2, 558 proteins were significantly associated at FDR <0.05 with risk of IHD (548 positively, 10 inversely) (Supplemental Table 3), with the top 5 positive protein associations being NT-proBNP (adjusted HR: 1.88; 95% CI: 1.67-2.11 per 1-SD higher concentration), WFDC2 (adjusted HR: 1.66; 95% CI: 1.46-1.88), pro-adrenomedullin (adjusted HR: 1.63; 95% CI: 1.41-1.89), EDA2R (adjusted HR: 1.62; 95% CI: 1.36-1.92), and GFRA1 (adjusted HR: 1.61; 95% CI: 1.42-1.82).

**Figure 2:**
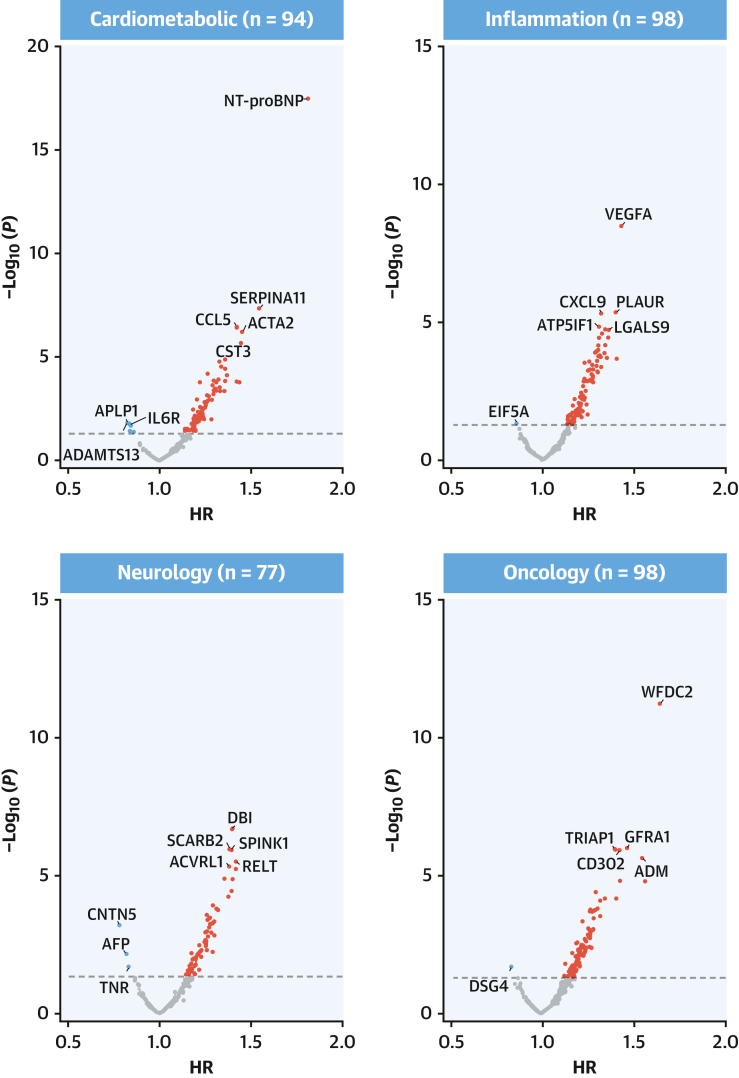
Adjusted HRs of IHD Associated With 1,463 Proteins in CKB The volcano plots show the associations of proteins with IHD stratified by OLINK panels in CKB after adjustment for confounding factors. All models were stratified by sex and region and adjusted for age, age^2^, fasting time, fasting time^2^, ambient temperature, ambient temperature^2^, plate identification, education, smoking, alcohol consumption, physical activity, systolic blood pressure (SBP), type 2 diabetes, ApoB/ApoA, and body mass index (BMI). Red, blue, and gray dots denote positive significant, inverse significant, and nonsignificant associations, respectively. Time in study was used as time scale in all models. Apo = apolipoprotein; other abbreviations as in Figure 1.

**Figure 3:**
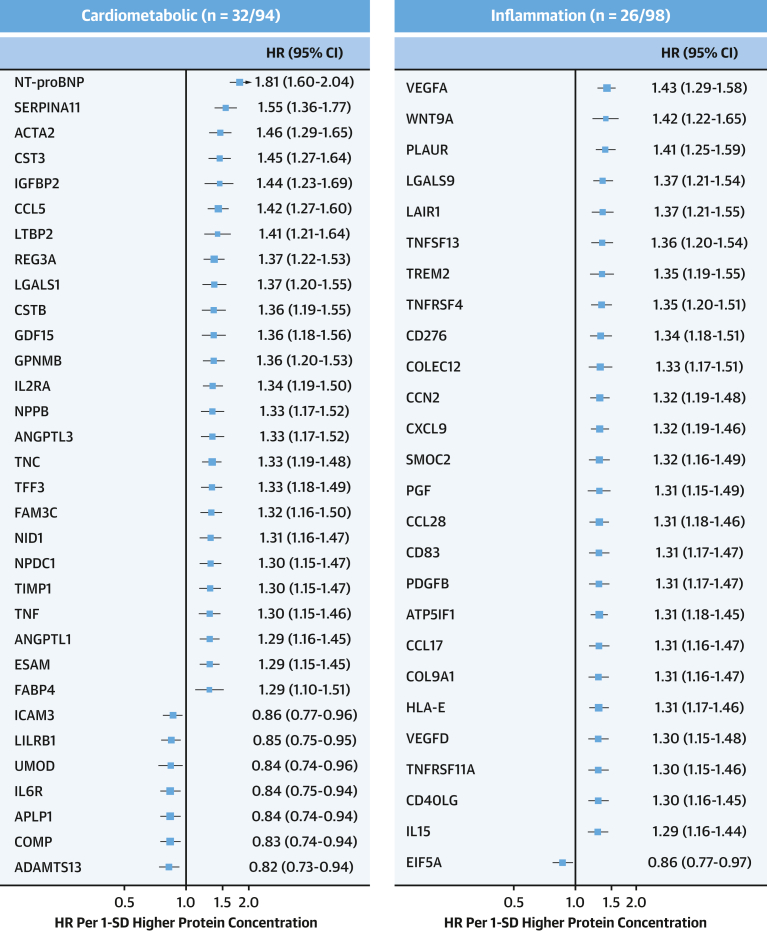

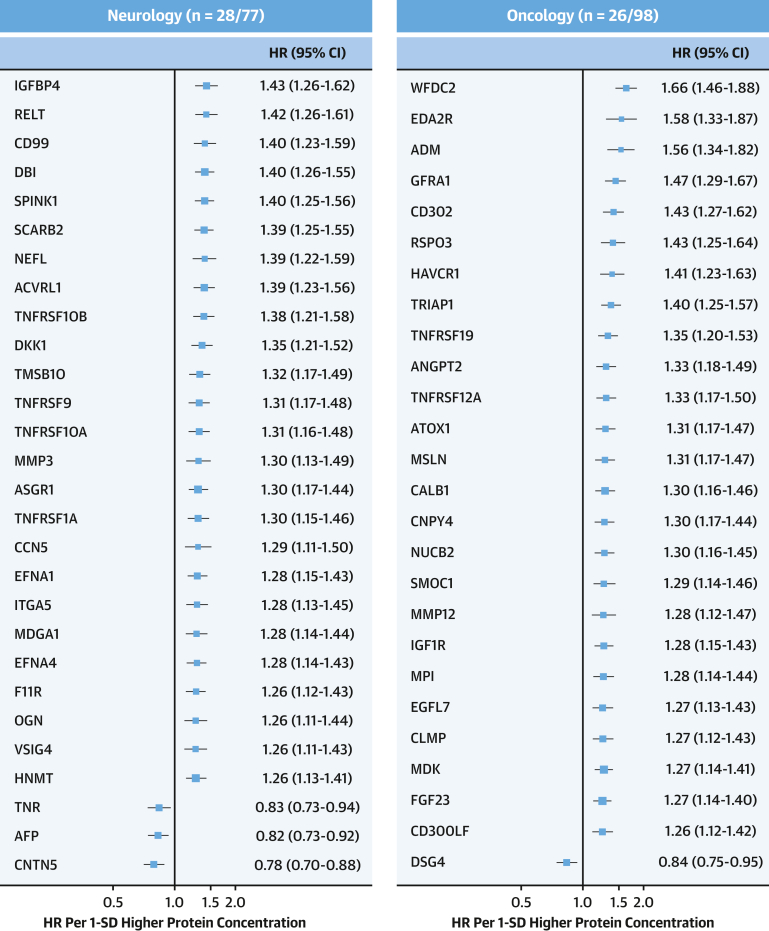
Adjusted HRs of IHD Associated With Selected Leading Proteins in CKB Forest plot shows adjusted HRs of IHD for proteins most strongly associated with IHD for cardiometabolic, inflammation, neurology, and oncology panels. The numbers of significantly associated proteins on each panel are shown and results for individual proteins are displayed. The models were adjusted for covariates as in Figure 2. The boxes are HRs and the horizontal lines are 95% CIs. The area of each box is inversely proportional to the variance of the log HR. Abbreviations as in Figure 1.

After sequential adjustment for SBP and diabetes, the number of significant protein associations decreased from 558 to 393 and 355, respectively, but with no further changes after additional adjustment for Apo (apolipoprotein) B/ApoA ratio and body mass index (Supplemental Table 3). Among the 361 significant protein associations in the final model, 349 were positive, including proprotein convertase subtilisin/kexin type 9 (PCSK9; adjusted HR: 1.23; 95% CI: 1.10-1.38), with little change in the effect sizes compared with model 2 for NT-proBNP (adjusted HR: 1.81; 95% CI: 1.60-2.04), WFDC2 (adjusted HR: 1.66; 95% CI: 1.46-1.88), EDA2R (adjusted HR: 1.58; 95% CI: 1.33-1.87), and pro-adrenomedullin (adjusted HR: 1.56; 95% CI: 1.34-1.82) (Supplemental Tables 3 and 4, Figures 2 and 3). These 361 proteins were only moderately correlated with each other, with 99.8% of protein pairs having correlation coefficients ranging from −0.5 to +0.5. After applying a more stringent Bonferroni-correction threshold, 93 proteins remained significantly associated with IHD (Supplemental Table 5).

The associations were typically log-linear, with no evidence of a threshold beyond which protein levels were no longer associated with IHD (Supplemental Figure 3). The results were unaltered in sensitivity analyses (Supplemental Table 6) or additional analyses without censoring any subcohort participants who developed incident IHD during follow-up (332 significant proteins in the final model, at FDR <0.05). In an internal replication analyses of subcohort participants, 236 proteins were significantly associated with risk of IHD (n = 232 cases) in the final model, with 147 (62%) proteins overlapping with those identified in the main analyses (Supplemental Table 7, Supplemental Figure 4).

### Genetic associations of proteins with IHD

Among the 361 significant proteins identified in CKB observational analyses, *cis*-pQTL variants were identified in CKB GWAS for 212. In 1-sample MR analyses, none of these associations were significant. In 2-sample MR analyses involving CC4D and Biobank Japan, 13 proteins (FURIN, Sortilin [SORT1], placenta growth factor [PGF], Asialoglycoprotein receptor 1 [ASGR1], C-C motif chemokine 17 [CCL17], angiopoietin-related protein 1 [ANGPTL1], proteinase-activated receptor 1 [F2R], OBP2B, TFPI, tenascin, EFNA1, REG1B, and matrix metalloproteinase [MMP]3) were significantly associated at FDR <0.05 with IHD (Table 2, Supplemental Figure 5). Of these 13 proteins, 3 (FURIN, SORT1, and PGF) had similar HRs in both observational and MR analyses, whereas the HRs for the remaining 10 proteins were less extreme in MR analyses albeit directionally concordant with the observational analyses (Supplemental Figure 6). Moreover, in 2-sample MR analyses solely involving Biobank Japan (n = 29,319 cases), 4 proteins (SORT, PGF, OBP2B, and ANGPTL1) were replicated (Table 2). The Pearson correlation coefficients of these 13 proteins with each other were all ≤0.75.

**Table 2:** Genetic Information and Key Findings in CC4D and Downstream Analyses for Proteins Associated With IHD in Europeans and Chinese

Protein Name	Gene Name	RNA Expression in Aorta^a^	Variant ID	MAF	Genetic Analysis		Drug Development			Target Development Level	
					Beta (SE)^a^	*P* Value	Drug Name	Outcomes	Trial Phase	Active Drug-Tclin^c^	Active Ligands-Tchem^d^
Europeans (UKB)											
FURIN	*FURIN*	Yes	15:91425232	0.32	0.306 (0.027)	5.69 × 10^-27^	—	—	—	0	289
PCSK9	*PCSK9*	Yes	1:55505647	0.01	0.220 (0.019)	1.02 × 10^-26^	Inclisiran	Cardiovascular disease	IV	3	62
TGFB1	*CCDC97*	Yes	19:41825191	0.17	0.219 (0.024)	6.66 × 10^-18^	Luspatercept	Anemia	IV	0	0
IL6R	*IL6R*	Yes	1:154426264	0.40	-0.031 (0.004)	9.85 × 10^-11^	Sarilumab	Rheumatoid arthritis	IV	3	0
BCAM	*BCAM*	Yes	19:45316588	0.03	0.141 (0.024)	2.86 × 10^-07^	—	—	—	0	0
COL6A3	*COL6A3*	Yes	2:238232752	0.60	0.263 (0.055)	6.25 × 10^-05^	Collagenase	Diabetic foot	IV	0	0
LTBP3	*LTBP3*	Yes	11:65319986	0.05	0.046 (0.011)	0.001	—	—	—	0	0
ANGPT1	*ZFPM2*	Yes	8:106581528	0.28	0.108 (0.028)	0.002	Trebananib	Peritoneal cancer	II	0	0
ITIH3	*ITIH3*	Yes	3:52828628	0.34	0.063 (0.016)	0.002	—	—	—	0	0
MMP3	*MMP3*	–	11:102713777	0.48	0.035 (0.010)	0.008	Marimastat	Lung cancer	II	0	1,352
ASGR1	*ASGR1*	–	17:7080316	0.19	0.114 (0.034)	0.010	Vupanorsen	Hypercholesterolemia	II	0	0
SCARB2	*SCARB2*	Yes	4:77097373	0.14	0.071 (0.022)	0.015	—	—	—	0	0
RARRES2	*RARRES2*	Yes	7:150039555	0.25	0.082 (0.026)	0.018	—	—	—	0	0
F2R	*F2R*	Yes	5:76028124	0.17	0.135 (0.043)	0.019	Vorapaxar Sulfate	Myocardial infarction	IV	1	362
TINAGL1	T*INAGL1*	Yes	1:32047305	0.61	0.188 (0.061)	0.043	—	—	—	0	0
CX3CL1	*CX3CL1*	Yes	16:57409062	0.61	0.071 (0.025)	0.045	Quetmolimab	Crohn's disease	II	0	0
Chinese (CKB)											
SORT1^b^	*CELSR2*	Yes	1:109274623	0.94	0.194 (0.014)	1.85 × 10^-37^	—	—	—	0	17
FURIN	*MAN2A2*	Yes	15:90897856	0.10	0.278 (0.024)	1.34 × 10^-27^	—	—	—	0	289
PGF^b^	*EIF2B2*	Yes	14:75002247	0.17	0.191 (0.026)	5.87 × 10^-12^	Aflibercept	Macular degeneration	IV	1	16
OBP2B^b^	*ABO*	–	9:133247439	0.20	0.063 (0.012)	6.15 × 10^-06^	—	—	—	0	0
ANGPTL1b	*RNA5SP69*	Yes	1:178603558	0.44	0.091 (0.019)	3.06 × 10^-05^	—	—	—	0	0
TFPI	*TFPI*	Yes	2:187467463	0.11	0.060 (0.014)	2 × 10^-03^	Andexanet alfa	Stroke	IV	0	501
CCL17	*CCL17*	–	5:76750290	0.50	0.094 (0.027)	8 × 10^-02^	—	—	—	0	0
F2R	*JMY*	Yes	16:57398911	0.51	0.082 (0.022)	0.003	Vorapaxar sulfate	Myocardial infarction	IV	1	362
REG1B	*REG1B*	–	2:79103043	0.09	0.034 (0.010)	0.013	—	—	—	0	0
ASGR1	*ASGR1*	–	11:102842889	0.33	0.130 (0.042)	0.020	Vupanorsen	Hypercholesterolemia	II	0	0
MMP3	*MMP3*	–	17:7176750	0.65	0.025 (0.008)	0.020	Marimastat	Lung cancer	III	0	1,352
EFNA1	*EFNA1*	Yes	1:155133578	0.17	0.048 (0.017)	0.032	—	—	—	0	0
TNC	*DEC1*	Yes	9:115144027	0.48	0.054 (0.019)	0.042	F16IL2	Merkel cell skin cancer	II	0	0

The proteins shown in bold were significantly associated with IHD in both Europeans and Chinese.

ANGPTL1 = angiopoietin-related protein 1; ASGR1 = Asialoglycoprotein receptor 1; CC4D = CARDIOGRAM+C4D; CCL17 = C-C motif chemokine 17; CKB = China Kadoorie Biobank; EFNA1 = ephrin-A1; F2R = proteinase-activated receptor 1; FURIN = furin; ID = identification; IHD = ischemic heart disease; MAF = minor allele frequency; MMP3 = matrix metalloproteinase-3; OBP2B = odorant-binding protein 2b; PGF = placenta growth factor; REG1B = lithostathine-1-beta; SORT1 = sortilin; Tchem = T-chemical; Tclin = T-clinical; TFPI = tissue factor pathway inhibitor; TNC = tenascin; UKB = UK Biobank.

a Beta and SE were calculated using Wald ratio formula.

b Additional replication in the Biobank Japan (29,319 IHD cases) and directionally consistent with observational analysed in CKB.

c Target has at least 1 approved drug.

d Target has at least 1 ChEMBL compound with an activity cutoff of <30 nmol/L.

**Figure 4:**
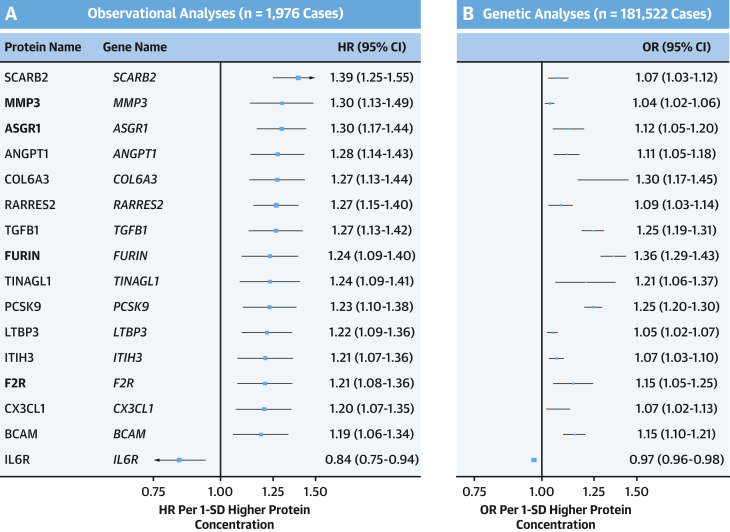
Replication of Observational Analyses in CKB in Genetic Analyses in CARDIOGRAM+C4D (A) The HRs (95% CI) of IHD per 1-SD higher protein concentration in CKB after adjustment for confounding factors. (B) The ORs (95% CI) of IHD in CARDIOGRAM+C4D per 1-SD higher protein concentration for these proteins in MR analyses. Symbols and conventions as in Figure 3.

In UKB, we found *cis*-pQTLs for 307 (85%) of the 361 IHD-associated proteins. In 2-sample MR analyses of these 307 *cis*-pQTLs, 16 showed significant associations with IHD risk (Figure 4, Table 2), with the directionally consistent, albeit less extreme effect sizes than those observed in CKB observational analyses (Supplemental Figure 7). These 16 proteins included 4 proteins (FURIN, MMP3, F2R, and ASGR1) identified in CKB, but most had different leading *cis*-pQTL variants and more extreme beta coefficients (Table 2, Figure 4). For SORT1, the OR was 1.09 (95% CI: 1.02-1.16), but this did not exceed the threshold for multiple testing (FDR-p = 0.068). For PCSK9, the *cis*-pQTL from UKB (rs11591147; MAF [minor allele frequency] = 0.01), but not from CKB (rs572512; MAF = 0.73), was strongly and positively associated with IHD (aHR: 1.25 [95% CI: 1.20-1.30] vs aHR: 0.94 [95% CI: 0.90-0.98]) (Figure 4). In contrast, *cis*-pQTLs for NT-proBNP in both CKB and UKB were unrelated to risk of IHD (CKB: 0.98, 95% CI: 0.94-1.02; UKB: 0.97, 95% CI: 0.93-1.02).

### Downstream analyses of novel proteins as potential drug targets for IHD

In phenome-wide association analyses of these 13 proteins, *cis*-pQTLs for FURIN were associated with several CVD traits, including high blood pressure and other CVD type traits, whereas SORT1 *cis*-pQTL was associated with 2 CVD types, lipids, and 7 additional traits (Supplemental Table 8). Likewise, *cis*-pQTLs for PGF, F2R, TFPI, and EFNA1 were related to CVD outcomes (PGF) and CVD risk factors. Within CKB, TFPI, PGF, F2R, and ASGR1 were each significantly associated with all 4 CVD risk factors (smoking, SBP, adiposity, and type 2 diabetes). Moreover, FURIN, ASGR1, SORT1, TFPI, PGF, F2R, and EFNA1 were also associated with SBP, adiposity, and type 2 diabetes.

Additional analyses of KO mouse models for these 13 proteins identified several CVD phenotypes (FURIN, TFPI, F2R, and EFNA1), adipose tissue (PGF), metabolism (OBP2B, TFPI, F2R, REG1B, ASGR1, ANGPTL1, and tenascin), and aging (TFPI, F2R, REG1B, and ASGR1) (Supplemental Table 9). In enrichment analysis using gene ontology terms, these proteins were enriched in 2 terms related to molecular function, nerve growth factor binding (SORT1 and FURIN; *P* = 0.0043; fold enrichment = 112.8) and neurotrophin binding (SORT1 and FURIN; *P* = 0.013; fold enrichment = 56.4) implicated in the pathogenesis of atherosclerosis. Analysis of chromatin enrichment using functional GWAS indicated that FURIN, PGF, and OBP2B were highly expressed, whereas CC17 and TFP1 were moderately expressed in endothelial cells. KEGG pathways of 361 proteins revealed the majority of proteins are involved in cytokine-cytokine receptor interaction (53 proteins; FDR-P = 5.24E-40), PI3K-Akt signaling pathway (34 proteins; FDR-P = 1.90E-16), and MAPK signaling pathway (26 proteins; FDR-P = 1.11E-11) (Supplemental Table 10).

Analysis of Open Targets and other databases indicated evidence of drug development, including phase II-IV trials, for 6 proteins (PGF, ASGR1, F2R, TFPI, tenascin, and MMP3), with 3 related to CVD outcomes or traits (F2R for MI, TFPI for ICH, and ASGR1 for familial hypercholesterolemia) (Table 2). However, there were no reports of drug targets for the 7 remaining proteins, including FURIN, CCL17, and SORT1. Drug target development for Tclin outcomes revealed PGF and F2R each having 1 “active drug,” whereas Tchem demonstrated “active ligands” for the majority of proteins including FURIN (Table 2).

## Discussion

Combined analyses of proteomic and genomic data in Chinese adults provided strong support for the causal relevance of 13 proteins for IHD, with 4 of these proteins (ie, FURIN, F2R, ASGR1, and MMP3) further replicated in European populations. Further downstream analyses such as phenome-wide associations and KO models confirmed the importance of several of these 13 proteins for CVD or CVD-related traits and their potentials as drug targets for IHD. Among these 13 proteins, there was, however, no evidence of drug development for 7, while for the remaining 6 proteins only 1 was associated with drug development for IHD. Moreover, none of them were identified in previous CC4D using large-scale GWAS data alone.^2^

Previous studies conducted in Western populations have reported inconsistent, or even conflicting, results for associations of plasma proteins with IHD,^2^^,^^4^^,^^15^^,^^24^ with the exception of NT-proBNP, vascular endothelial growth factor, IL-6, IL-8, and PCSK9, which have been consistently associated with IHD.^15^^,^^25^, ^26^, ^27^ Indeed, NT-proBNP was the single protein most strongly associated with IHD in almost all studies (including the present study), but few previous studies assessed the causal relevance of these associations. The present study involving both CKB and UKB showed that *cis*-pQTLs for NT-proBNP were unrelated with IHD. This suggested that NT-proBNP is not a causal risk factor for IHD, but a marker of subclinical atherosclerosis, which could inform risk prediction but not treatment. For PCSK9, we did not find significant genetic associations with IHD using leading *cis-*pQTL in CKB. However, separate analyses of >100,000 genotyped participants in CKB demonstrated that higher levels of *PCSK9* genetic scores were significantly and positively associated with risk of CVD (manuscript under review).

Recently, a genetic study of 304 proteins measured using mass spectrometry among 2,410 Chinese adults reported significant associations of *cis*-QTLs for 3 proteins (apolipoprotein a, apolipoprotein E, and haptoglobin) with CAD in 2-sample MR analyses involving Biobank Japan. However, none of these proteins overlapped with those evaluated in CKB. Moreover, unlike in the present report, that Chinese study did not include observational analyses of proteins with CVD outcomes.^28^ In a previous report from the DECODE study of 35,559 individuals, 1,287 of 4,907 proteins measured using SomaScan platform were significantly associated with myocardial infarction (MI) (n = 3,457 cases), with the top 3 proteins being NPPB (HR: 1.64 per 1-SD higher concentration), MMP-12 (1.55), and CTHRC1 (1.50).^9^ The present study also showed significant associations of NPPB and MMP-12 with IHD (1.41 and 1.35, respectively), but did not assess CTHRC1. Likewise, one of the proteins that was most strongly and inversely associated with IHD (contactin-5) in the present study was also reported by DECODE (HR: 0.78 vs 0.77). However, DECODE did not evaluate the causal relevance of these associations using MR analyses.^9^ To date, no large studies have directly compared associations of overlapping proteins measured using different assay platforms (eg, OLINK EXPLORE vs SomaScan) with IHD in the same individuals. Nevertheless, it has been reported that the available OLINK panels include more targeted selection of proteins, whereas SomaScan panels include greater analytical breath.^29^

The integrated analyses of proteomic and genomic data in the present study demonstrated that FURIN (also known as PCSK3) was the most strongly (and causally) IHD-associated protein. The *FURIN* gene encodes the subtilisin-like proprotein convertase (PCSK3). FURIN plays a regulatory role in inflammation and atherosclerosis.^30^ FURIN is also involved in activation of proBNP, PCSK9, and ANGPTL, which each contribute to the development of atherosclerosis.^31^ In addition, FURIN has been implicated in the regulation of blood pressure by controlling activation of prorenin receptors, proliferation of vascular smooth muscle, and activation of epithelial sodium channels. In experimental KO models, irreversible inhibition of FURIN (via α1-antitrypsin) attenuates the progression of atherosclerosis.^32^ Previous studies reported that higher levels of FURIN were associated with MCP-1, leading to activation of inflammation.^33^ Despite the latter associations with FURIN, there has been little previous evidence linking FURIN with incident IHD in population studies. In a 3-year follow-up of 1,100 individuals with acute MI, plasma levels of FURIN were unrelated with risk of recurrent CVD outcomes after MI.^30^ Previous studies in Europeans reported associations of IHD with tyrosine-protein kinase (FES) that is high-linkage disequilibrium with FURIN. If confirmed, FES could explain in part the previously reported genetic association of *FURIN* with IHD or with hypertension.^34^^,^^35^ However, the present study demonstrated no associations of FES with IHD (HR: 1.09; *P* = 0.260) or with SBP (beta: 0.021; *P* = 0.091). Overall, the available evidence provides strong support for *FURIN* as a potential novel target for IHD, but we found no drugs targeting *FURIN* for the treatment of IHD.

For the 3 remaining proteins (ie, F2R, ASGR1, and MMP3), we found evidence of drug development. F2R is a G protein–coupled receptor that is irreversibly activated by MMP proteins.^36^ Previous studies have reported higher levels of F2R expression in human atherosclerotic lesions.^37^ Inhibition of F2R receptors by vorapaxar, which reduces thrombin-induced platelet activation after coronary revascularization in patients with acute occlusion of coronary arteries,^38^ has been approved for treatment of IHD, stroke, and other CVD outcomes. ASGR1 is associated with low-density lipoprotein–cholesterol and is a target for treatment of familial hypercholesterolemia.^39^ A previous genetic study in Europeans (42,524 cases and 249,414 controls) reported that ASGR1 insufficiency was associated with lower levels of low-density lipoprotein–cholesterol and lower risks of IHD,^40^ consistent with present study findings. MMP proteins are highly expressed in endothelial and vascular smooth muscle cells and have been implicated in angiogenesis and atherosclerosis.^41^ In the DECODE study of 35,559 Icelanders with 3,457 incident MI cases, plasma levels of MMP3, assayed using the SomaScan platform, were inversely associated with risk of MI (HR: 0.86), but the cause-effect nature of association was not evaluated.^9^ In contrast, we demonstrated positive associations of MMP3 with risks of IHD in both observational and genetic analyses. It is unclear whether the discrepant results for MMP3 between the DECODE and CKB studies reflect assay differences or other ethnic differences between 2 populations. Importantly, MMP3 is currently being investigated as a novel protein target for treatment of lung and breast cancer, highlighting potential opportunities for drug-repurposing for IHD.

Among the remaining 9 proteins causally associated with IHD in Chinese but not in Europeans, there was no evidence of drug development for SORT1, OBP2B, ANGPTL1, CCL17, REG1B, and EFNA1, whereas none of the remaining 3 proteins (PGF, TFPI, and TNC) were associated with drug development for IHD. Both ANGPTL and angiopoietin-1 proteins have been implicated in angiogenesis, inflammation, lipids, and glucose metabolism.^42^ ANGPT1 is an established drug target for the treatment of cancer, but we did not identify any evidence of drug development related to IHD.^43^ Hence, given the present study findings, ANGPT1 should also be considered as a protein target for IHD. SORT1 augments PCSK9 secretion,^44^ and has been implicated in development of atherosclerosis.^45^ Previous studies have provided genetic support, consistent with present study findings, for the causal relevance of SORT1 for MI and other CVD outcomes.^46^ However, we found no evidence of drug development for SORT1 for any CVD outcomes. CCL17 is a chemokine that is selectively expressed in human macrophages and plays a pivotal role in atherosclerosis by promotion of leukocyte infiltration into endothelial cells.^47^ We found somewhat stronger genetic associations for CCL17 with IHD in CKB than in UKB (1.10 vs 1.04), which may reflect differences in the lead variants used or their MAFs (UKB 0.08 vs CKB 0.50) between the 2 populations. In animal experiments, KO of *CCL17* genes delayed progression of atherosclerosis and attenuated ischemia-reperfusion injury.^48^ Importantly, CCL17 is also a potential novel treatment target for age-related diseases, cardiac hypertrophy, and heart failure.^49^ Two previous small observational studies reported positive associations of CCL17 with IHD independent of established CVD risk factors.^50^ These, together with similar findings in the present study, provided strong support for CCL17 as a novel protein target for treatment of IHD.

### Study limitations

First, it was not possible to obtain independent replication of the observational analyses in other East Asian populations due to the lack of available data. However, internal replication with incident cases in the subcohort and demonstration of significant associations for several proteins known to be associated with IHD (eg, NT-proBNP, NPPB, MMP-12, and PCSK9) confirmed the validity of the findings of observational analyses. Second, the present 1-sample MR analyses in CKB lacked statistical power to evaluate the causal relevance of the protein associations identified in observational analyses. Third, the 2-sample MR analyses were chiefly based on European ancestry populations. Nevertheless, further analyses using different *cis*-pQTLs identified in the UKB replicated the causal associations of 4 proteins with IHD. Fourth, we applied multiple testing correction at all stages of analyses, which, although appropriate, could have theoretically resulted in overcorrection and false-negative findings. Indeed, in 2-sample MR analyses without additional correction for multiple testing, a total of 11, rather than 4, proteins were causally associated with IHD in both CKB and UKB.

## Conclusions

Integrated analyses of proteomics with genomic data provided causal support for FURIN and multiple other proteins as potential novel drug targets for treatment of IHD. Previous studies demonstrated that support from genomic data for exposures with disease outcomes improved their likelihood of success in subsequent drug development trials.^51^^,^^52^ The present study identified 13 potential novel protein targets for drug treatment of IHD that had not been previously discovered using large-scale genomic data alone.^2^ In addition to discovery of potential novel drug targets, large-scale proteomics and genetic data in diverse populations should improve risk prediction, early detection, and prevention strategies of IHD and many other diseases.

Perspectives **COMPETENCY IN MEDICAL KNOWLEDGE:** Integrated analyses of proteomic and genetic data in prospective studies or diverse populations identify FURIN and other proteins as novel potential targets for treatment of IHD.

**TRANSLATIONAL OUTLOOK:** Future research will expand the array of potential drug targets that can be evaluated in clinical studies of a diversity of patients with IHD.

## Funding Support and Author Disclosures

The CKB baseline survey and the first resurvey were supported by the Kadoorie Charitable Foundation in Hong Kong. The long-term follow-up and subsequent resurveys have been supported by Wellcome grants to Oxford University (212946/Z/18/Z, 202922/Z/16/Z, 104085/Z/14/Z, 088158/Z/09/Z) and grants from the National Natural Science Foundation of China (82192901, 82192904, 82192900) and from the National Key Research and Development Program of China (2016YFC0900500). The UK Medical Research Council (MC_UU_00017/1, MC_UU_12026/2, MC_U137686851), Cancer Research UK (C16077/A29186, C500/A16896), and British Heart Foundation (CH/1996001/9454) provide core funding to the Clinical Trial Service Unit and Epidemiological Studies Unit, Oxford University for the project. The proteomic assays were supported by a BHF Intermediate Clinical Research Fellowship to MVH (FS/18/23/33512), Novo Nordisk, and OLINK. DNA extraction and genotyping were supported by GlaxoSmithKline and the UK Medical Research Council (MC-PC-13049, MC-PC-14135). Dr Holmes is currently employed by 23andMe (and owns stock in 23andMe, Inc). Dr Howson is a full-time employee of Novo Nordisk Research Centre Oxford Limited and owns shares in Novo Nordisk. All other authors have reported that they have no relationships relevant to the contents of this paper to disclose.
