# Supplementary material for: Plasma Proteomics to Identify Drug Targets for Ischemic Heart Disease
Source: J Am Coll Cardiol. 2023 Nov 14;82(20):1906–20. doi: 10.1016/j.jacc.2023.09.804 (PMC10641761; doi:10.1016/j.jacc.2023.09.804)
Supplement: Supplemental Material [file mmc1.docx]

**Supplementary material**

**“**Plasma Proteomics to Identify Drug Targets for Ischemic Heart Disease**” by M Mazidi et al.**

**Table of contents**

[Members of the China Kadoorie Biobank Collaborative Group 3](#_Toc141697105)

[Supplementary methods 4](#_Toc141697106)

[eTable 1: Number of quality control (QC) and assay warnings per assay, by OLINK panels 7](#_Toc141697107)

[eTable 2: Proportions of samples with values below level of detection (LOD) per assay, by OLINK panels 8](#_Toc141697108)

[eTable 3: Number of proteins significantly associated at FDR<0.05 with risk of IHD after step-wise adjustment for conventional CVD risk factors in observational analyses, by OLINK panel 9](#_Toc141697109)

[eTable 4: Mean (SD) levels and hazard ratios (SE) for IHD for 361 significantly associated proteins 10](#_Toc141697110)

[eTable 5: Number of proteins significantly associated at Bonferroni-corrected threshold with risk of IHD after step-wise adjustment for conventional CVD risk factors 14](#_Toc141697111)

[eTable 6: Number of proteins significantly associated at FDR<0.05 with risk of IHD in the final model after exclusion of proteins with assay warning and below level of detection (LOD), by OLINK panel 15](#_Toc141697112)

[eTable 7: Number of proteins significantly associated at FDR<0.05 with risk of IHD after step-wise adjustment for conventional CVD risk factors in observational analyses among sub-cohort participants, by OLINK panel 16](#_Toc141697113)

[eTable 8: Results of phenome-wide association studies for 13 candidate causal proteins 17](#_Toc141697114)

[eTable 9: Specific phenotypes identified from single-gene KO mouse models for individual proteins 18](#_Toc141697115)

[eTable 10: Top 10 KEGG pathways for 361 significantly associated proteins for IHD 19](#_Toc141697116)

[eFigure 1: Distribution of concentration levels of 12 proteins showing bimodal distribution pattern 20](#_Toc141697117)

[eFigure 2: Total number and name of the top 10 proteins significantly associated at FDR<0.05 with major CVD risk factors in observational analyses 21](#_Toc141697118)

[eFigure 3A: Adjusted HRs for risk of IHD by quartiles of top 25 proteins in observational analyses, in OLINK Cardiometabolic panel 22](#_Toc141697119)

[eFigure 3B: Adjusted HRs for risk of IHD by quartiles of top 25 proteins in observational analyses, in OLINK Inflammation panel 23](#_Toc141697120)

[eFigure 3C: Adjusted HRs for risk of IHD by quartiles of top 25 proteins in observational analyses, in OLINK Neurology panel 24](#_Toc141697121)

[eFigure 3D: Adjusted HRs for risk of IHD by quartiles of top 25 proteins in observational analyses, in OLINK Oncology panel 25](#_Toc141697122)

[eFigure 4: Total number of the proteins significantly associated at FDR<0.05 with risk of IHD in case sub-cohort vs. sub-cohort only, in final model of observational analyses 26](#_Toc141697123)

[26](#_Toc141697124)

[eFigure 5: Global agreement in effect estimates between observational (CKB) and genetic (CKB & CC4D) analyses 27](#_Toc141697125)

[eFigure 6: Adjusted HRs and ORs for risk of IHD associated with 1 SD higher levels of 13 significant proteins in (a) observational (CKB) and (b) genetic (CC4D) analyses, respectively 28](#_Toc141697126)

[eFigure 7: Global agreement in effect estimates between observational (CKB) and genetic (CC4D & UKB) analyses 29](#_Toc141697127)

### Members of the China Kadoorie Biobank Collaborative Group

**International Steering Committee:** Junshi Chen, Zhengming Chen (PI), Robert Clarke, Rory Collins, Liming Li (PI), Chen Wang, Jun Lv, Richard Peto, Robin Walters.

**International Co-ordinating Centre, Oxford:** Daniel Avery, Maxim Barnard, Derrick Bennett, Ruth Boxall, Sushila Burgess, Ka Hung Chan, Yiping Chen, Zhengming Chen, Johnathan Clarke, Robert Clarke, Huaidong Du, Ahmed Edris Mohamed, Hannah Fry, Simon Gilbert, Pek Kei Im, Andri Iona, Maria Kakkoura, Christiana Kartsonaki, Hubert Lam, Kuang Lin, James Liu, Mohsen Mazidi, Iona Millwood, Sam Morris, Qunhua Nie, Alfred Pozarickij, Paul Ryder, Saredo Said, Dan Schmidt, Becky Stevens, Iain Turnbull, Robin Walters, Baihan Wang, Lin Wang, Neil Wright, Ling Yang, Xiaoming Yang, Pang Yao.

**National Co-ordinating Centre, Beijing:** Xiao Han, Can Hou, Qingmei Xia, Chao Liu, Jun Lv, Pei, Dianjanyi Sun, Canqing Yu

**10 Regional Co-ordinating Centres:**

**Guangxi** Provincial CDC: Naying Chen, Duo Liu, Zhenzhu Tang. Liuzhou CDC: Ningyu Chen, Qilian Jiang, Jian Lan, Mingqiang Li, Yun Liu, Fanwen Meng, Jinhuai Meng, Rong Pan, Yulu Qin, Ping Wang, Sisi Wang, Liuping Wei, Liyuan Zhou. **Gansu** Provincial CDC: Caixia Dong, Pengfei Ge, Xiaolan Ren. Maiji CDC: Zhongxiao Li, Enke Mao, Tao Wang, Hui Zhang, Xi Zhang. **Hainan** Provincial CDC: Jinyan Chen, Ximin Hu, Xiaohuan Wang. Meilan CDC: Zhendong Guo, Huimei Li, Yilei Li, Min Weng, Shukuan Wu. **Heilongjiang** Provincial CDC: Shichun Yan, Mingyuan Zou, Xue Zhou. Nangang CDC: Ziyan Guo, Quan Kang, Yanjie Li, Bo Yu, Qinai Xu. **Henan** Provincial CDC: Liang Chang, Lei Fan, Shixian Feng, Ding Zhang, Gang Zhou. Huixian CDC: Yulian Gao, Tianyou He, Pan He, Chen Hu, Huarong Sun, Xukui Zhang. **Hunan** Provincial CDC: Biyun Chen, Zhongxi Fu, Yuelong Huang, Huilin Liu, Qiaohua Xu, Li Yin. Liuyang CDC: Huajun Long, Xin Xu, Hao Zhang, Libo Zhang. **Jiangsu** Provincial CDC: Jian Su, Ran Tao, Ming Wu, Jie Yang, Jinyi Zhou, Yonglin Zhou. Suzhou CDC: Yihe Hu, Yujie Hua, Jianrong Jin, Fang Liu, Jingchao Liu, Yan Lu, Liangcai Ma, Aiyu Tang, Jun Zhang. **Qingdao** CDC: Liang Cheng, Ranran Du, Ruqin Gao, Feifei Li, Shanpeng Li, Yongmei Liu, Feng Ning, Zengchang Pang, Xiaohui Sun, Xiaocao Tian, Shaojie Wang, Yaoming Zhai, Hua Zhang, Licang CDC: Wei Hou, Silu Lv, Junzheng Wang. **Sichuan** Provincial CDC: Xiaofang Chen, Xianping Wu, Ningmei Zhang, Weiwei Zhou. Pengzhou CDC: Xiaofang Chen, Jianguo Li, Jiaqiu Liu, Guojin Luo, Qiang Sun, Xunfu Zhong. **Zhejiang** Provincial CDC: Weiwei Gong, Ruying Hu, Hao Wang,Meng Wang, Min Yu. Tongxiang CDC: Lingli Chen, Qijun Gu, Dongxia Pan，Chunmei Wang, Kaixu Xie, Xiaoyi Zhang.

# Supplementary methods

*Proteomics assays*

Stored baseline plasma samples for IHD cases and subcohort participants were retrieved, thawed, and aliquoted into 96-well plates (with 8 wells per plate reserved for QC assays), with each plate containing both cases and controls in varying proportions, plated in the order that they were retrieved from storage at the Wolfson laboratory in Oxford. The plates were shipped on dry ice to the OLINK Laboratory at Uppsala, Sweden, for analysis using the OLINK EXPLORE platform, which included four similar-sized panels (i.e. cardiometabolic, inflammation, neurology and oncology).(1) Plasma levels of these proteins were normalized using both internal and inter-plate controls and all were transformed using a pre-determined correction factor. The limit of detection (LOD) was determined using negative control samples (buffers without antigens). The levels of plasma proteins were provided in NPX units on a logarithmic scale (whereby a high NPX indicated a high protein concentration).

Samples were flagged for quality control (QC) warnings if incubation controls deviated by ≥0.3 from the median values for all samples on any plate and any proteins with QC warnings observations with such warnings were excluded from subsequent analyses. Individual samples were flagged for assay warnings if values deviated by 3-fold from negative controls. Proteins with assay warnings or values below the LOD were only excluded in sensitivity but not the main analyses. The number of QC or sample warnings per assay in addition to proportions with values below LOD are shown in **eTables 1 and 2**.

Among a total of 1,472 proteins measured, three proteins (CXCL8, IL6, TNF) were replicated across all four panels, yielding 1463 unique proteins. The distribution of correlation coefficients between all proteins indicated that 98.8% had correlation coefficients (r) ranging between -0.5 to +0.5, and 12 proteins had a bimodal distribution (**eFigure 1**). The within-assay correlation coefficients for the three replicating proteins varied from 0.95-0.99 for CXCL8, 0.90-0.94 for TNF and 0.75-0.81 for IL6.

*Statistical methods*

Plasma protein levels (NPX) were standardized (i.e. values were divided by their SD) and analysed as continuous variables. The associations of proteins with established cardiovascular disease (CVD) risk factors (e.g. SBP, BMI, diabetes and smoking) were examined using linear regression, adjusted for sex, study region, age and age^2^, time since last meal and its square, ambient temperature and its square, and plate ID.

In observational analyses, Cox regression models were used to estimate adjusted HRs (and 95% CIs) for IHD using the Prentice pseudo-partial likelihood for case-cohort designs.(2) The sub-cohort participants who developed incident IHD during follow-up were censored at time of IHD diagnosis. All analyses were stratified by sex and the 10 study areas, and adjusted for: (i) age, age^2^, time since last meal and its square, ambient temperature and its square, and plate ID (*Model 1*); (ii) additionally for education (five categories), smoking (four categories), alcohol drinking (six categories), and physical activity (MET-h) (*Model 2*); (iii) additionally for systolic blood pressure (SBP) (*Model 3*); (iv) additionally for diabetes (yes/no; *Model 4*); (v) additionally for ApoB/ApoA ratio (*Model 5*); and (vi) additionally for body mass index (BMI) (*Model 6)*. For proteins significantly associated with IHD in Model 6, we further examined the shape of the associations by quartiles of individual proteins.

In sensitivity analyses, we further adjusted for history of chronic kidney disease (CKD), excluded values with assay warnings or values below the LOD, and did not censor sub-cohort participants who developed IHD during follow-up (n=232 cases). Replication of the observational associations included separate prospective associations of proteins with incident IHD events recorded in the sub-cohort participants.

In one- and two-sample MR analyses, we used *cis*-pQTLs derived from GWAS of protein levels in Chinese and European adults to assess the causal relevance for IHD of significant protein associations in CKB observational analyses. In both Chinese (Said et al, Manuscript in Preparation) and Europeans,(3) *cis-*pQTLs were identified as genome-wide significant (*P*<5×10^-8^) association signals where the lead variants were within 500 Kb of the protein structural gene.

For proteins with *cis*-pQTLs identified in CKB, we undertook one-sample MR analyses in the case-cohort sample (n=1976 cases) or in the overall CKB genotyping dataset (n=6499 cases).(4) Firstly, protein levels were regressed on SNPs and the observed covariates. Secondly, the associations with IHD cases were assessed using logistic regression on the predicted levels of plasma proteins identified. The coefficients of IHD for the predicted proteins from the second stage represent the genetically predicted causal effects per 1 SD higher plasma levels of individual proteins for risk of IHD.

# eTable 1: Number of quality control (QC) and assay warnings per assay, by OLINK panels

| **OLINK panels** | **Number of individuals with warnings per assay** | | | | | |
| --- | --- | --- | --- | --- | --- | --- |
|  | **0-49** | | **50-99** | | **100-200** | |
|  | **Assay** | **QC** | **Assay** | **QC** | **Assay** | **QC** |
| **Cardiometabolic** | 321 | 184 | 16 | 114 | 9 | 71 |
| **Inflammation** | 347 | 178 | 15 | 110 | 5 | 80 |
| **Neurology** | 340 | 156 | 11 | 0 | 8 | 211 |
| **Oncology** | 349 | 148 | 6 | 110 | 1 | 110 |

# eTable 2: Proportions of samples with values below level of detection (LOD) per assay, by OLINK panels

| **Proportions of values below LOD per assay** | **OLINK panels** | | | | | |
| --- | --- | --- | --- | --- | --- | --- |
|  | **Cardiometabolic** | **Inflammation** | **Neurology** | **Oncology** | | **Overall** |
| **0.0 - 0.24** | 331 | 302 | 282 | 289 | 1204 | |
| **0.25 - 0.49** | 8 | 15 | 25 | 25 | 73 | |
| **0.50 - 0.74** | 16 | 16 | 18 | 27 | 77 | |
| **0.75 - 1.0** | 14 | 35 | 42 | 27 | 118 | |
| Counts are the number of assays which have a proportion in that bin. | | | | | | |

# eTable 3: Number of proteins significantly associated at FDR<0.05 with risk of IHD after step-wise adjustment for conventional CVD risk factors in observational analyses, by OLINK panel

| **Models** | **OLINK panels** | | | | **All (Inverse)** |
| --- | --- | --- | --- | --- | --- |
|  | **Cardiometabolic** | **Inflammation** | **Neurology** | **Oncology** |  |
| Basic model* | 128 | 128 | 111 | 136 | 503 (9) |
| + Education, smoking, alcohol, physical activity | 141 | 151 | 119 | 147 | 558 (10) |
| + SBP | 100 | 108 | 84 | 101 | 393 (10) |
| + Diabetes | 90 | 98 | 73 | 94 | 355 (11) |
| + ApoB/ApoA ratio† | 93 | 98 | 73 | 94 | 358 (12) |
| + BMI | 93 | 97 | 75 | 96 | 361 (12) |
| * Basic model: Age, age², fasting time, fasting time², ambient temperature, ambient temperature² and plate ID.  † Not included in the existing OLINK Explore platform, but measured in other way among the participants  Abbreviations: FDR=False discovery rate; CVD=Cardiovascular disease; SBP=Systolic blood pressure; BMI=Body mass index. | | | | | |

| eTable 4: Mean (SD) levels and hazard ratios (SE) for IHD for 361 significantly associated proteins | | | | | | | | | | | | | |
| --- | --- | --- | --- | --- | --- | --- | --- | --- | --- | --- | --- | --- | --- |
| **ID** | **Name of protein** | **HR** | **SE** | **FDR** | **Mean concentration** | **SD of concentration** | **ID** | **Name of protein** | **HR** | **SE** | **FDR** | **Mean concentration** | **SD of concentration** |
| 1 | NTproBNP | 1.808 | 0.062 | 2.52E-18 | 5.081 | 17.926 | 182 | EIF4G1 | 1.159 | 0.056 | 0.035529 | 1.141 | 1.836 |
| 2 | SERPINA11 | 1.552 | 0.068 | 3.18E-08 | 2.564 | 0.883 | 183 | FABP1 | 1.181 | 0.063 | 0.037066 | 3.039 | 4.983 |
| 3 | ACTA2 | 1.463 | 0.062 | 2.89E-07 | 2.01 | 1.942 | 184 | PPP1R9B | 1.148 | 0.053 | 0.037918 | 0.729 | 0.771 |
| 4 | CCL5 | 1.423 | 0.059 | 3.41E-07 | 3.68 | 9.25 | 185 | SCRN1 | 1.143 | 0.051 | 0.039903 | 0.955 | 0.862 |
| 5 | CST3 | 1.447 | 0.065 | 1.57E-06 | 1.314 | 0.407 | 186 | NPPC | 1.173 | 0.061 | 0.040424 | 1.918 | 1.095 |
| 6 | REG3A | 1.366 | 0.059 | 9.42E-06 | 2.098 | 1.616 | 187 | MANF | 1.158 | 0.057 | 0.040651 | 2.112 | 7.286 |
| 7 | TNC | 1.327 | 0.056 | 1.97E-05 | 1.545 | 0.67 | 188 | EIF5A | 0.865 | 0.058 | 0.047364 | 1.022 | 0.433 |
| 8 | IL2RA | 1.338 | 0.058 | 3.18E-05 | 1.313 | 0.512 | 189 | LGMN | 1.145 | 0.054 | 0.048447 | 1.336 | 0.479 |
| 9 | GPNMB | 1.355 | 0.062 | 3.96E-05 | 1.454 | 0.392 | 190 | TNFSF12 | 1.16 | 0.059 | 0.049525 | 1.629 | 0.429 |
| 10 | RARRES2 | 1.269 | 0.049 | 4.79E-05 | 1.086 | 1.777 | 191 | DBI | 1.398 | 0.054 | 1.93E-07 | 1.275 | 1.186 |
| 11 | LGALS1 | 1.365 | 0.066 | 8.27E-05 | 1.52 | 0.477 | 192 | SCARB2 | 1.394 | 0.056 | 4.60E-07 | 1.386 | 0.726 |
| 12 | TFF3 | 1.326 | 0.06 | 8.27E-05 | 1.558 | 1.039 | 193 | SPINK1 | 1.396 | 0.058 | 8.11E-07 | 1.647 | 0.782 |
| 13 | IGFBP2 | 1.443 | 0.081 | 0.00015 | 2.037 | 1.277 | 194 | RELT | 1.423 | 0.063 | 2.16E-06 | 1.256 | 0.406 |
| 14 | CCL14 | 1.222 | 0.044 | 0.00015 | 1.626 | 2.019 | 195 | ACVRL1 | 1.387 | 0.06 | 3.58E-06 | 1.454 | 0.428 |
| 15 | ANGPTL1 | 1.294 | 0.057 | 0.00015 | 1.259 | 0.343 | 196 | IGFBP4 | 1.425 | 0.065 | 3.71E-06 | 1.544 | 0.965 |
| 16 | CSTB | 1.357 | 0.067 | 0.000155 | 1.464 | 0.812 | 197 | DKK1 | 1.353 | 0.058 | 1.20E-05 | 1.734 | 1.124 |
| 17 | NID1 | 1.308 | 0.06 | 0.000169 | 1.433 | 0.405 | 198 | CD99 | 1.398 | 0.064 | 1.20E-05 | 1.184 | 0.56 |
| 18 | LTBP2 | 1.41 | 0.078 | 0.000213 | 2.182 | 0.878 | 199 | NEFL | 1.394 | 0.067 | 3.18E-05 | 2.293 | 3.376 |
| 19 | ESAM | 1.294 | 0.058 | 0.000214 | 1.429 | 0.429 | 200 | TNFRSF10B | 1.383 | 0.066 | 4.50E-05 | 1.681 | 0.63 |
| 20 | NPPB | 1.332 | 0.066 | 0.000269 | 3.537 | 12.197 | 201 | ASGR1 | 1.298 | 0.054 | 5.71E-05 | 1.142 | 0.399 |
| 21 | GDF15 | 1.356 | 0.071 | 0.00035 | 2.383 | 1.709 | 202 | TNFRSF9 | 1.313 | 0.059 | 0.000131 | 1.244 | 0.408 |
| 22 | FAM3C | 1.319 | 0.065 | 0.000374 | 1.387 | 0.472 | 203 | TMSB10 | 1.319 | 0.061 | 0.000154 | 1.477 | 1.155 |
| 23 | TNF | 1.296 | 0.061 | 0.000375 | 1.611 | 0.614 | 204 | TXNDC5 | 1.261 | 0.053 | 0.000235 | 1.704 | 1.42 |
| 24 | TIMP1 | 1.303 | 0.062 | 0.00039 | 1.426 | 0.418 | 205 | EFNA1 | 1.283 | 0.057 | 0.000235 | 1.208 | 0.415 |
| 25 | ANGPTL3 | 1.331 | 0.068 | 0.000425 | 1.372 | 0.408 | 206 | TNFRSF10A | 1.308 | 0.063 | 0.00039 | 1.551 | 0.76 |
| 26 | NPDC1 | 1.303 | 0.063 | 0.000445 | 1.69 | 0.676 | 207 | TNFRSF1A | 1.297 | 0.062 | 0.000425 | 1.42 | 0.509 |
| 27 | REG1A | 1.266 | 0.057 | 0.000559 | 2.16 | 1.847 | 208 | APP | 1.253 | 0.054 | 0.000499 | 1.941 | 1.42 |
| 28 | LGALS3 | 1.267 | 0.059 | 0.000912 | 1.438 | 0.367 | 209 | MDGA1 | 1.278 | 0.059 | 0.000539 | 1.99 | 1.253 |
| 29 | COL6A3 | 1.274 | 0.061 | 0.001021 | 1.202 | 0.469 | 210 | CNTN5 | 0.783 | 0.059 | 0.000543 | 1.528 | 0.486 |
| 30 | HSPG2 | 1.29 | 0.064 | 0.001021 | 1.276 | 0.37 | 211 | EFNA4 | 1.276 | 0.059 | 0.00059 | 1.35 | 0.481 |
| 31 | IL6 | 1.206 | 0.048 | 0.001241 | 1.63 | 5.768 | 212 | HNMT | 1.262 | 0.058 | 0.000791 | 1.714 | 1.43 |
| 32 | THPO | 1.255 | 0.059 | 0.001345 | 1.193 | 0.429 | 213 | ITGA5 | 1.279 | 0.063 | 0.001117 | 1.266 | 0.286 |
| 33 | TNFSF13B | 1.251 | 0.059 | 0.001485 | 1.328 | 0.344 | 214 | NID2 | 1.247 | 0.057 | 0.001254 | 1.517 | 0.872 |
| 34 | PDGFA | 1.252 | 0.059 | 0.001609 | 2.316 | 2.848 | 215 | JAM2 | 1.256 | 0.06 | 0.001557 | 1.393 | 0.409 |
| 35 | RETN | 1.24 | 0.057 | 0.001609 | 1.718 | 0.965 | 216 | F11R | 1.264 | 0.062 | 0.001609 | 1.361 | 0.436 |
| 36 | TYMP | 1.238 | 0.056 | 0.001617 | 1.087 | 0.615 | 217 | MMP3 | 1.3 | 0.07 | 0.001719 | 1.797 | 1.451 |
| 37 | SERPINE1 | 1.256 | 0.061 | 0.001831 | 1.432 | 1.119 | 218 | CD74 | 1.248 | 0.059 | 0.002016 | 1.311 | 0.457 |
| 38 | PRSS2 | 1.258 | 0.061 | 0.001919 | 1.4 | 1.182 | 219 | LAYN | 1.261 | 0.064 | 0.002849 | 2.043 | 1.055 |
| 39 | CLEC5A | 1.253 | 0.062 | 0.002629 | 1.387 | 0.412 | 220 | VSIG4 | 1.262 | 0.064 | 0.002929 | 1.555 | 0.874 |
| 40 | ICAM5 | 1.221 | 0.055 | 0.002929 | 1.375 | 0.567 | 221 | RSPO1 | 1.254 | 0.063 | 0.003033 | 2.06 | 1.054 |
| 41 | PCSK9 | 1.233 | 0.058 | 0.003017 | 0.948 | 0.27 | 222 | FKBP5 | 1.215 | 0.055 | 0.003313 | 1.347 | 1.898 |
| 42 | SCARF1 | 1.239 | 0.06 | 0.003163 | 1.314 | 0.371 | 223 | OGN | 1.263 | 0.067 | 0.004189 | 1.541 | 0.792 |
| 43 | PLIN3 | 1.241 | 0.06 | 0.003163 | 1.198 | 0.418 | 224 | CLEC14A | 1.238 | 0.061 | 0.004189 | 1.354 | 0.472 |
| 44 | TFPI | 1.185 | 0.047 | 0.003191 | 1.313 | 1.539 | 225 | TNFRSF1B | 1.231 | 0.06 | 0.004383 | 1.463 | 0.522 |
| 45 | PTGDS | 1.232 | 0.059 | 0.004102 | 1.404 | 0.479 | 226 | CCN5 | 1.293 | 0.076 | 0.005397 | 1.602 | 0.67 |
| 46 | IGFBP6 | 1.242 | 0.062 | 0.004286 | 1.421 | 0.551 | 227 | CD99L2 | 1.191 | 0.053 | 0.007176 | 1.228 | 0.263 |
| 47 | CLUL1 | 1.208 | 0.054 | 0.004336 | 1.169 | 0.559 | 228 | IL1R1 | 1.23 | 0.063 | 0.007657 | 1.418 | 0.322 |
| 48 | SELP | 1.231 | 0.06 | 0.004336 | 1.076 | 0.39 | 229 | TNFRSF6B | 1.209 | 0.058 | 0.007775 | 1.466 | 1.812 |
| 49 | ITGB1BP2 | 1.225 | 0.059 | 0.00437 | 1.517 | 3.378 | 230 | CLEC1B | 1.213 | 0.059 | 0.007775 | 1.583 | 1.236 |
| 50 | CXCL5 | 1.229 | 0.061 | 0.005514 | 7.445 | 21.591 | 231 | AFP | 0.821 | 0.061 | 0.007775 | 1.354 | 1.009 |
| 51 | MCFD2 | 1.217 | 0.058 | 0.005514 | 1.42 | 0.458 | 232 | FABP5 | 1.187 | 0.053 | 0.008761 | 1.146 | 1.704 |
| 52 | ANG | 1.196 | 0.053 | 0.005657 | 1.052 | 0.358 | 233 | NDRG1 | 1.186 | 0.053 | 0.008796 | 1.606 | 7.137 |
| 53 | SPON2 | 1.242 | 0.065 | 0.006501 | 1.47 | 0.504 | 234 | MFGE8 | 1.199 | 0.057 | 0.009008 | 1.458 | 0.677 |
| 54 | COL18A1 | 1.251 | 0.067 | 0.006755 | 1.197 | 0.256 | 235 | SERPINB9 | 1.178 | 0.053 | 0.011355 | 1.738 | 0.876 |
| 55 | SPP1 | 1.226 | 0.062 | 0.007383 | 2.487 | 1.115 | 236 | CST5 | 1.199 | 0.058 | 0.011432 | 1.631 | 1.046 |
| 56 | NECTIN2 | 1.227 | 0.063 | 0.007851 | 1.464 | 0.491 | 237 | CX3CL1 | 1.204 | 0.06 | 0.013195 | 1.34 | 0.497 |
| 57 | PI3 | 1.21 | 0.059 | 0.008819 | 0.997 | 0.705 | 238 | IL1RAP | 1.197 | 0.06 | 0.016056 | 1.34 | 0.321 |
| 58 | REG1B | 1.212 | 0.06 | 0.008834 | 2.129 | 2.033 | 239 | OBP2B | 1.198 | 0.061 | 0.016355 | 1.772 | 2.093 |
| 59 | TINAGL1 | 1.236 | 0.066 | 0.009019 | 1.524 | 0.348 | 240 | PIK3IP1 | 1.193 | 0.059 | 0.016541 | 1.491 | 0.556 |
| 60 | FABP4 | 1.291 | 0.08 | 0.009161 | 1.039 | 0.752 | 241 | CD164 | 1.165 | 0.052 | 0.017755 | 1.193 | 1.78 |
| 61 | DEFA1-DEFA1B | 1.194 | 0.056 | 0.009684 | 0.997 | 0.615 | 242 | SUMF2 | 1.192 | 0.06 | 0.017755 | 1.408 | 0.302 |
| 62 | SPARCL1 | 1.186 | 0.054 | 0.010127 | 1.48 | 0.308 | 243 | FUT8 | 1.17 | 0.054 | 0.017956 | 1.585 | 0.793 |
| 63 | VWF | 1.195 | 0.056 | 0.010456 | 1.143 | 0.575 | 244 | SEMA4D | 1.189 | 0.06 | 0.020025 | 1.129 | 0.241 |
| 64 | IL1RL1 | 1.204 | 0.059 | 0.010467 | 1.101 | 0.557 | 245 | TNR | 0.832 | 0.064 | 0.0206 | 1.408 | 0.53 |
| 65 | LDLR | 1.207 | 0.06 | 0.010745 | 1.413 | 0.709 | 246 | DKK4 | 1.171 | 0.055 | 0.021274 | 1.081 | 0.652 |
| 66 | ITIH3 | 1.21 | 0.061 | 0.010818 | 1.165 | 0.417 | 247 | BCAM | 1.188 | 0.06 | 0.021274 | 1.362 | 0.323 |
| 67 | CD59 | 1.221 | 0.064 | 0.010965 | 1.268 | 0.334 | 248 | VWC2 | 1.179 | 0.058 | 0.02383 | 1.944 | 1.333 |
| 68 | CDH2 | 1.244 | 0.07 | 0.011276 | 2.148 | 0.861 | 249 | MSR1 | 1.216 | 0.07 | 0.023969 | 2.355 | 1.03 |
| 69 | LACTB2 | 1.182 | 0.055 | 0.014011 | 1.337 | 0.711 | 250 | ULBP2 | 1.175 | 0.058 | 0.025114 | 1.801 | 0.905 |
| 70 | IL6R | 0.838 | 0.058 | 0.014048 | 1.129 | 0.305 | 251 | GOLM2 | 1.169 | 0.056 | 0.027312 | 1.423 | 0.303 |
| 71 | COMP | 0.833 | 0.06 | 0.014401 | 1.568 | 0.505 | 252 | CRTAM | 1.183 | 0.061 | 0.028095 | 1.759 | 0.691 |
| 72 | IGSF8 | 1.202 | 0.061 | 0.014494 | 1.333 | 0.388 | 253 | MESD | 1.153 | 0.052 | 0.028145 | 1.195 | 2.353 |
| 73 | AHCY | 1.185 | 0.056 | 0.014724 | 1.153 | 0.867 | 254 | NGF | 1.156 | 0.053 | 0.029525 | 1.075 | 0.167 |
| 74 | CLEC1A | 1.182 | 0.055 | 0.014724 | 1.404 | 0.813 | 255 | PTPRN2 | 1.17 | 0.059 | 0.033314 | 1.259 | 0.481 |
| 75 | IL6ST | 1.207 | 0.063 | 0.015144 | 1.161 | 0.18 | 256 | SNCG | 1.201 | 0.068 | 0.03334 | 1.844 | 1.363 |
| 76 | PPP1R2 | 1.185 | 0.057 | 0.015748 | 1.042 | 0.74 | 257 | CRIP2 | 1.161 | 0.056 | 0.035532 | 1.702 | 1.454 |
| 77 | ADAMTS13 | 0.825 | 0.065 | 0.015975 | 1.116 | 0.229 | 258 | LIF | 1.141 | 0.05 | 0.036959 | 1.064 | 1.226 |
| 78 | APLP1 | 0.836 | 0.06 | 0.016541 | 2.647 | 1.55 | 259 | TBCB | 1.159 | 0.057 | 0.03844 | 1.915 | 4.212 |
| 79 | RNASET2 | 1.195 | 0.061 | 0.017644 | 1.423 | 0.315 | 260 | TBCC | 1.162 | 0.058 | 0.039135 | 1.106 | 1.385 |
| 80 | SORT1 | 1.174 | 0.055 | 0.018277 | 1.158 | 0.363 | 261 | SERPINB6 | 1.151 | 0.055 | 0.042595 | 1.375 | 0.477 |
| 81 | LILRB1 | 0.846 | 0.059 | 0.023388 | 1.011 | 0.261 | 262 | NOS1 | 1.183 | 0.066 | 0.043838 | 1.073 | 0.672 |
| 82 | CA13 | 1.18 | 0.059 | 0.025125 | 1.153 | 2.322 | 263 | GDNF | 1.151 | 0.055 | 0.046182 | 1.397 | 0.781 |
| 83 | IGFBP7 | 1.152 | 0.052 | 0.028957 | 1.626 | 1.434 | 264 | PIGR | 1.158 | 0.059 | 0.048447 | 1.626 | 0.543 |
| 84 | AOC3 | 1.19 | 0.064 | 0.030812 | 1.658 | 0.391 | 265 | FCER2 | 1.143 | 0.053 | 0.049438 | 1.415 | 0.995 |
| 85 | CEBPB | 1.132 | 0.046 | 0.033314 | 1.178 | 0.583 | 266 | WFDC2 | 1.657 | 0.064 | 1.66E-12 | 2.206 | 0.645 |
| 86 | CEACAM8 | 1.159 | 0.056 | 0.035043 | 1.604 | 0.784 | 267 | GFRA1 | 1.47 | 0.065 | 6.54E-07 | 1.35 | 0.558 |
| 87 | CRX | 1.144 | 0.051 | 0.035711 | 5.538 | 20.851 | 268 | CD302 | 1.433 | 0.062 | 8.11E-07 | 1.913 | 0.67 |
| 88 | CCL27 | 1.2 | 0.069 | 0.036959 | 1.395 | 0.825 | 269 | TRIAP1 | 1.403 | 0.058 | 8.11E-07 | 1.29 | 0.785 |
| 89 | UMOD | 0.84 | 0.066 | 0.036959 | 1.397 | 0.585 | 270 | ADM | 1.564 | 0.078 | 1.22E-06 | 2.186 | 0.655 |
| 90 | STK4 | 1.16 | 0.057 | 0.037844 | 1.018 | 0.852 | 271 | EDA2R | 1.576 | 0.088 | 1.46E-05 | 1.967 | 1.113 |
| 91 | CXCL16 | 1.181 | 0.064 | 0.041097 | 0.97 | 0.193 | 272 | RSPO3 | 1.428 | 0.069 | 1.70E-05 | 1.853 | 0.701 |
| 92 | ICAM3 | 0.861 | 0.058 | 0.041097 | 1.263 | 0.291 | 273 | CNPY4 | 1.299 | 0.053 | 3.96E-05 | 1.233 | 0.677 |
| 93 | CCL18 | 1.141 | 0.052 | 0.046151 | 1.361 | 3.174 | 274 | TNFRSF19 | 1.352 | 0.062 | 4.79E-05 | 1.347 | 0.632 |
| 94 | PLXNB3 | 1.157 | 0.058 | 0.047226 | 1.047 | 0.261 | 275 | HAVCR1 | 1.414 | 0.072 | 5.26E-05 | 1.303 | 3.983 |
| 95 | VEGFA | 1.429 | 0.052 | 4.81E-09 | 1.775 | 1.073 | 276 | ANGPT2 | 1.325 | 0.06 | 8.13E-05 | 1.157 | 0.462 |
| 96 | PLAUR | 1.408 | 0.062 | 3.06E-06 | 1.534 | 0.543 | 277 | CALB1 | 1.304 | 0.058 | 0.000131 | 1.466 | 0.74 |
| 97 | CXCL9 | 1.321 | 0.052 | 5.24E-06 | 2.612 | 3.718 | 278 | MSLN | 1.308 | 0.059 | 0.000134 | 1.747 | 1.354 |
| 98 | ATP5IF1 | 1.311 | 0.052 | 1.21E-05 | 1.24 | 2.552 | 279 | ATOX1 | 1.309 | 0.059 | 0.00015 | 1.552 | 1.192 |
| 99 | LGALS9 | 1.367 | 0.061 | 1.80E-05 | 1.432 | 0.465 | 280 | MDK | 1.268 | 0.053 | 0.000169 | 1.53 | 1.809 |
| 100 | TNFRSF4 | 1.346 | 0.058 | 1.80E-05 | 1.432 | 0.557 | 281 | IGF1R | 1.282 | 0.055 | 0.00017 | 1.33 | 0.354 |
| 101 | CCN2 | 1.324 | 0.056 | 3.18E-05 | 1.652 | 1.02 | 282 | NUCB2 | 1.296 | 0.058 | 0.00017 | 1.277 | 0.391 |
| 102 | LAIR1 | 1.367 | 0.063 | 3.18E-05 | 1.436 | 0.571 | 283 | FGF23 | 1.266 | 0.053 | 0.000206 | 1.313 | 4.083 |
| 103 | CCL28 | 1.312 | 0.055 | 3.79E-05 | 2.144 | 1.991 | 284 | TNFRSF12A | 1.325 | 0.064 | 0.000235 | 1.234 | 0.465 |
| 104 | TNFSF13 | 1.357 | 0.063 | 4.79E-05 | 1.442 | 0.408 | 285 | MPI | 1.281 | 0.058 | 0.000353 | 0.795 | 0.563 |
| 105 | HLA-E | 1.305 | 0.056 | 5.82E-05 | 1.489 | 0.611 | 286 | RAD23B | 1.219 | 0.047 | 0.000463 | 0.595 | 0.529 |
| 106 | CD83 | 1.312 | 0.057 | 8.13E-05 | 1.438 | 0.705 | 287 | SMOC1 | 1.29 | 0.063 | 0.000788 | 1.481 | 0.639 |
| 107 | CD40LG | 1.299 | 0.056 | 8.27E-05 | 1.432 | 1.313 | 288 | SPARC | 1.253 | 0.056 | 0.000912 | 2.213 | 1.807 |
| 108 | CD276 | 1.338 | 0.063 | 0.000121 | 1.479 | 0.481 | 289 | AREG | 1.258 | 0.058 | 0.001097 | 1.287 | 0.683 |
| 109 | IL15 | 1.293 | 0.056 | 0.000126 | 1.207 | 0.32 | 290 | RBP5 | 1.262 | 0.059 | 0.001117 | 1.794 | 1.585 |
| 110 | PDGFB | 1.311 | 0.06 | 0.00015 | 2.448 | 2.672 | 291 | EGFL7 | 1.269 | 0.061 | 0.001201 | 3.124 | 1.959 |
| 111 | WNT9A | 1.417 | 0.077 | 0.000167 | 1.763 | 1.179 | 292 | CCN4 | 1.256 | 0.059 | 0.001335 | 1.366 | 0.651 |
| 112 | CCL17 | 1.308 | 0.06 | 0.000169 | 1.924 | 2.636 | 293 | C4BPB | 1.245 | 0.057 | 0.001378 | 1.451 | 0.563 |
| 113 | TREM2 | 1.355 | 0.068 | 0.00017 | 2.392 | 1.392 | 294 | CD300LF | 1.264 | 0.061 | 0.001437 | 2 | 1.059 |
| 114 | COL9A1 | 1.306 | 0.06 | 0.000174 | 0.778 | 0.412 | 295 | CLMP | 1.268 | 0.062 | 0.00148 | 1.142 | 0.289 |
| 115 | SMOC2 | 1.315 | 0.062 | 0.000216 | 1.25 | 0.519 | 296 | SLAMF8 | 1.258 | 0.06 | 0.00161 | 1.612 | 0.921 |
| 116 | TNFRSF11A | 1.3 | 0.061 | 0.000294 | 1.751 | 0.939 | 297 | PVALB | 1.242 | 0.059 | 0.002275 | 1.454 | 1.574 |
| 117 | COLEC12 | 1.329 | 0.066 | 0.0003 | 1.424 | 0.438 | 298 | NTF4 | 1.198 | 0.049 | 0.002559 | 1.35 | 3.743 |
| 118 | CXCL1 | 1.255 | 0.053 | 0.000339 | 3.564 | 13 | 299 | KLK11 | 1.23 | 0.057 | 0.002936 | 1.318 | 0.469 |
| 119 | LAT | 1.275 | 0.057 | 0.000374 | 2.28 | 2.259 | 300 | LTBP3 | 1.219 | 0.055 | 0.003076 | 1.418 | 0.938 |
| 120 | MMP1 | 1.264 | 0.056 | 0.000463 | 7.554 | 7.167 | 301 | MMP12 | 1.282 | 0.07 | 0.003265 | 1.967 | 1.542 |
| 121 | PGF | 1.313 | 0.066 | 0.000527 | 1.512 | 0.457 | 302 | FAM3B | 1.243 | 0.061 | 0.003481 | 1.258 | 0.481 |
| 122 | PREB | 1.251 | 0.054 | 0.000543 | 1.129 | 0.623 | 303 | NBL1 | 1.246 | 0.062 | 0.003552 | 1.383 | 0.425 |
| 123 | ANGPT1 | 1.276 | 0.059 | 0.000543 | 2.928 | 3.013 | 304 | HSPB6 | 1.262 | 0.067 | 0.004283 | 1.117 | 0.6 |
| 124 | VEGFD | 1.301 | 0.064 | 0.000605 | 0.955 | 0.361 | 305 | TAFA5 | 1.245 | 0.063 | 0.004286 | 1.63 | 0.704 |
| 125 | ANGPTL4 | 1.28 | 0.06 | 0.000627 | 1.337 | 0.577 | 306 | VEGFC | 1.218 | 0.057 | 0.004286 | 1.839 | 1.33 |
| 126 | TGFB1 | 1.268 | 0.059 | 0.000791 | 1.723 | 0.796 | 307 | GCNT1 | 1.208 | 0.054 | 0.004286 | 1.644 | 1.417 |
| 127 | CKAP4 | 1.284 | 0.062 | 0.000791 | 1.485 | 0.541 | 308 | FCRLB | 1.211 | 0.056 | 0.005039 | 2.009 | 5.062 |
| 128 | BTN3A2 | 1.235 | 0.054 | 0.001087 | 1.852 | 0.992 | 309 | CPXM1 | 1.199 | 0.053 | 0.005246 | 1.872 | 1.403 |
| 129 | IL7 | 1.231 | 0.053 | 0.001117 | 1.636 | 1.813 | 310 | FURIN | 1.236 | 0.062 | 0.005397 | 1.164 | 0.412 |
| 130 | CCL7 | 1.263 | 0.06 | 0.001156 | 1.591 | 1.523 | 311 | TFPI2 | 1.201 | 0.055 | 0.006946 | 0.893 | 1.078 |
| 131 | METAP1D | 1.235 | 0.054 | 0.001201 | 1.39 | 1.831 | 312 | FXN | 1.196 | 0.054 | 0.006951 | 1.192 | 0.784 |
| 132 | LILRB4 | 1.282 | 0.064 | 0.001345 | 3.427 | 1.461 | 313 | CDKN1A | 1.213 | 0.058 | 0.006957 | 1.403 | 2.838 |
| 133 | SPINT2 | 1.254 | 0.059 | 0.001345 | 2.2 | 0.703 | 314 | BTC | 1.167 | 0.047 | 0.007176 | 0.52 | 3.099 |
| 134 | EGF | 1.242 | 0.057 | 0.001609 | 0.843 | 1.3 | 315 | CD38 | 1.232 | 0.063 | 0.007331 | 1.786 | 0.62 |
| 135 | STX8 | 1.24 | 0.057 | 0.00161 | 0.91 | 0.551 | 316 | LTA4H | 1.23 | 0.063 | 0.007804 | 0.382 | 0.267 |
| 136 | TIMP3 | 1.225 | 0.055 | 0.002277 | 3.418 | 6.171 | 317 | F3 | 1.226 | 0.062 | 0.007824 | 1.329 | 0.436 |
| 137 | MATN2 | 1.238 | 0.06 | 0.003163 | 1.525 | 0.416 | 318 | HBEGF | 1.199 | 0.057 | 0.008981 | 1.806 | 0.52 |
| 138 | HPCAL1 | 1.21 | 0.054 | 0.00369 | 1.486 | 1.159 | 319 | CEACAM3 | 1.185 | 0.053 | 0.009019 | 1.249 | 1.665 |
| 139 | SPINK4 | 1.236 | 0.061 | 0.004383 | 1.439 | 1.009 | 320 | KLK13 | 1.205 | 0.059 | 0.010331 | 2.797 | 2.7 |
| 140 | TNFRSF14 | 1.236 | 0.061 | 0.004406 | 1.441 | 0.483 | 321 | CLEC6A | 1.195 | 0.056 | 0.010331 | 1.141 | 0.596 |
| 141 | CD70 | 1.229 | 0.06 | 0.004903 | 1.261 | 0.532 | 322 | PRTG | 1.201 | 0.058 | 0.010547 | 1.341 | 0.571 |
| 142 | MMP10 | 1.211 | 0.056 | 0.004903 | 1.462 | 0.894 | 323 | IQGAP2 | 1.192 | 0.056 | 0.011276 | 1.133 | 0.965 |
| 143 | PLA2G4A | 1.204 | 0.054 | 0.005114 | 1.504 | 1.979 | 324 | BAMBI | 1.204 | 0.061 | 0.014312 | 1.182 | 0.439 |
| 144 | IL24 | 1.182 | 0.049 | 0.005122 | 1.177 | 1.41 | 325 | PDCD1LG2 | 1.198 | 0.06 | 0.014401 | 1.291 | 0.518 |
| 145 | CLEC7A | 1.232 | 0.061 | 0.005242 | 1.276 | 0.61 | 326 | CAPG | 1.209 | 0.063 | 0.014809 | 0.699 | 0.462 |
| 146 | CXCL3 | 1.211 | 0.056 | 0.005375 | 3.333 | 4.343 | 327 | FGFBP1 | 1.2 | 0.061 | 0.015118 | 1.305 | 0.508 |
| 147 | CLIP2 | 1.206 | 0.057 | 0.007657 | 1.596 | 5.622 | 328 | P4HB | 1.206 | 0.062 | 0.015446 | 1.174 | 0.373 |
| 148 | CXCL10 | 1.2 | 0.056 | 0.007824 | 0.953 | 1.032 | 329 | DRG2 | 1.183 | 0.056 | 0.015725 | 1.103 | 0.613 |
| 149 | CDSN | 1.217 | 0.061 | 0.008713 | 1.348 | 0.74 | 330 | HGS | 1.17 | 0.053 | 0.016541 | 1.196 | 1.233 |
| 150 | PRKAB1 | 1.171 | 0.049 | 0.008761 | 1.262 | 4.05 | 331 | PPM1A | 1.131 | 0.042 | 0.017755 | 1.257 | 1.879 |
| 151 | SPON1 | 1.246 | 0.068 | 0.008761 | 1.438 | 0.69 | 332 | SORCS2 | 1.219 | 0.067 | 0.017882 | 2.025 | 1.293 |
| 152 | PRDX5 | 1.204 | 0.058 | 0.009082 | 0.58 | 0.628 | 333 | LRRC25 | 1.191 | 0.06 | 0.01792 | 1.545 | 1.624 |
| 153 | F2R | 1.208 | 0.059 | 0.009144 | 1.981 | 0.781 | 334 | CFC1 | 1.184 | 0.058 | 0.018639 | 1.45 | 0.811 |
| 154 | MLN | 1.217 | 0.062 | 0.010073 | 3.512 | 3.274 | 335 | SERPINA9 | 1.186 | 0.059 | 0.020851 | 1.602 | 1.242 |
| 155 | KLRD1 | 1.195 | 0.057 | 0.011544 | 1.491 | 0.744 | 336 | SCG2 | 1.199 | 0.063 | 0.021274 | 1.735 | 0.627 |
| 156 | CCL13 | 1.202 | 0.06 | 0.012886 | 1.61 | 1.27 | 337 | CCL8 | 1.162 | 0.052 | 0.021274 | 1.493 | 1.222 |
| 157 | CCL23 | 1.193 | 0.058 | 0.013866 | 1.055 | 0.41 | 338 | AMBP | 1.2 | 0.064 | 0.022709 | 1.293 | 0.478 |
| 158 | SCGB1A1 | 1.207 | 0.062 | 0.01438 | 1.488 | 0.794 | 339 | STX4 | 1.177 | 0.058 | 0.023969 | 1.201 | 0.553 |
| 159 | AGRN | 1.203 | 0.061 | 0.014401 | 1.283 | 0.526 | 340 | MAVS | 1.157 | 0.052 | 0.025381 | 1.146 | 0.913 |
| 160 | LY6D | 1.193 | 0.059 | 0.014724 | 1.204 | 0.857 | 341 | DSG4 | 0.842 | 0.061 | 0.025415 | 1.335 | 1.113 |
| 161 | SLC39A5 | 1.183 | 0.056 | 0.014809 | 1.091 | 2.141 | 342 | MSRA | 1.164 | 0.055 | 0.028193 | 0.825 | 0.611 |
| 162 | CEACAM21 | 1.168 | 0.052 | 0.014809 | 0.673 | 0.533 | 343 | CDNF | 1.177 | 0.06 | 0.028791 | 1.699 | 0.705 |
| 163 | TFF2 | 1.208 | 0.063 | 0.015184 | 2.336 | 1.502 | 344 | CD300E | 1.176 | 0.059 | 0.028791 | 1.399 | 0.665 |
| 164 | FSTL3 | 1.225 | 0.069 | 0.016541 | 1.312 | 0.51 | 345 | DAB2 | 1.165 | 0.056 | 0.02891 | 1.286 | 2.276 |
| 165 | IL18 | 1.184 | 0.057 | 0.017644 | 0.776 | 0.965 | 346 | EPHA2 | 1.181 | 0.061 | 0.030886 | 1.339 | 0.573 |
| 166 | NUDC | 1.183 | 0.057 | 0.01791 | 1.095 | 0.679 | 347 | DPY30 | 1.175 | 0.06 | 0.030886 | 1.005 | 1.008 |
| 167 | GOPC | 1.172 | 0.055 | 0.019174 | 1.31 | 2.099 | 348 | LGALS7-LGALS7B | 1.162 | 0.056 | 0.034386 | 1.409 | 0.685 |
| 168 | MGLL | 1.19 | 0.06 | 0.019592 | 1.281 | 1.755 | 349 | PSMA1 | 1.154 | 0.054 | 0.035711 | 1.088 | 1.161 |
| 169 | CD40 | 1.193 | 0.061 | 0.020703 | 1.469 | 0.48 | 350 | ADAMTS15 | 1.184 | 0.064 | 0.036877 | 4.578 | 2.057 |
| 170 | CST7 | 1.153 | 0.05 | 0.021665 | 1.475 | 1.673 | 351 | NINJ1 | 1.132 | 0.047 | 0.036959 | 1.208 | 2.438 |
| 171 | LAMP3 | 1.214 | 0.068 | 0.022441 | 1.62 | 0.968 | 352 | KLK10 | 1.15 | 0.053 | 0.036959 | 1.141 | 0.538 |
| 172 | CXCL17 | 1.248 | 0.078 | 0.022709 | 2.474 | 1.721 | 353 | KIR3DL1 | 1.145 | 0.052 | 0.036959 | 1.699 | 4.281 |
| 173 | MPIG6B | 1.2 | 0.064 | 0.022709 | 1.698 | 2.053 | 354 | PDCD1 | 1.162 | 0.058 | 0.038617 | 1.507 | 0.622 |
| 174 | MERTK | 1.185 | 0.061 | 0.025114 | 1.917 | 0.648 | 355 | TGFBR2 | 1.171 | 0.061 | 0.039484 | 1.205 | 0.48 |
| 175 | BCL2L11 | 1.183 | 0.06 | 0.025381 | 1.878 | 0.658 | 356 | XCL1 | 1.151 | 0.054 | 0.040835 | 2.012 | 1.916 |
| 176 | TGFA | 1.16 | 0.054 | 0.027038 | 0.968 | 0.362 | 357 | CEP20 | 1.149 | 0.054 | 0.041097 | 0.843 | 1.941 |
| 177 | CCL25 | 1.181 | 0.061 | 0.028868 | 1.363 | 0.632 | 358 | RTBDN | 1.173 | 0.062 | 0.041706 | 1.579 | 0.52 |
| 178 | HLA-DRA | 1.143 | 0.049 | 0.028989 | 2.162 | 2.807 | 359 | DCBLD2 | 1.174 | 0.062 | 0.04244 | 1.818 | 0.562 |
| 179 | BTN2A1 | 1.177 | 0.06 | 0.029525 | 1.317 | 0.369 | 360 | ENTPD2 | 1.152 | 0.055 | 0.044283 | 1.204 | 0.602 |
| 180 | TNFRSF11B | 1.215 | 0.071 | 0.029525 | 1.383 | 0.568 | 361 | CD207 | 1.165 | 0.061 | 0.049391 | 1.042 | 0.65 |
| 181 | LAMA4 | 1.167 | 0.058 | 0.034386 | 1.346 | 0.359 |  |  |  |  |  |  |  |

# eTable 5: Number of proteins significantly associated at Bonferroni-corrected threshold with risk of IHD after step-wise adjustment for conventional CVD risk factors

| **Models** | **OLINK panels** | | | | **All (Inverse)** |
| --- | --- | --- | --- | --- | --- |
|  | **Cardiometabolic** | **Inflammation** | **Neurology** | **Oncology** |  |
| Basic model* | 39 | 26 | 27 | 26 | 118 (1) |
| + Education, smoking, alcohol, physical activity | 48 | 42 | 30 | 36 | 156 (1) |
| + SBP | 26 | 32 | 24 | 25 | 107 (1) |
| + Diabetes | 27 | 28 | 18 | 20 | 93 (0) |
| + ApoB/ApoA ratio† | 26 | 27 | 18 | 20 | 91 (0) |
| + BMI | 26 | 28 | 18 | 21 | 93 (0) |
| * Basic model: Age, age², fasting time, fasting time², ambient temperature, ambient temperature² and plate ID.  † Not included in the current OLINK Explore panel but measured in other ways among study participants  Abbreviations: CVD=Cardiovascular disease; SBP=Systolic blood pressure; BMI=Body mass index | | | | | |

# eTable 6: Number of proteins significantly associated at FDR<0.05 with risk of IHD in the final model after exclusion of proteins with assay warning and below level of detection (LOD), by OLINK panel

| **Exclusion** | **OLINK Panels** | | | | **All** |
| --- | --- | --- | --- | --- | --- |
|  | **Cardiometabolic** | **Inflammation** | **Neurology** | **Oncology** |  |
| **a). Assay warning** | 94 | 98 | 75 | 96 | 363 |
|  |  |  |  |  |  |
| **b). Below LOD** | 93 | 103 | 84 | 95 | 375 |
|  |  |  |  |  |  |
| **c). Both** | 93 | 100 | 88 | 95 | 376 |
| Adjustment in the final Model*: Age, age², fasting time, fasting time², ambient temperature, ambient temperature², plate ID, education, smoking, alcohol, physical activity, SBP, diabetes, ApoB/ApoA ratio and BMI. | | | | | |

# eTable 7: Number of proteins significantly associated at FDR<0.05 with risk of IHD after step-wise adjustment for conventional CVD risk factors in observational analyses among sub-cohort participants, by OLINK panel

| **Models** | **OLINK panels** | | | | **All (Inverse)** |
| --- | --- | --- | --- | --- | --- |
|  | **Cardiometabolic** | **Inflammation** | **Neurology** | **Oncology** |  |
| Basic model* | 68 | 64 | 79 | 78 | 289 (4) |
| + Education, smoking, alcohol, physical activity | 65 | 69 | 73 | 76 | 283 (4) |
| + SBP | 54 | 59 | 57 | 61 | 231 (3) |
| + Diabetes | 54 | 59 | 57 | 62 | 232 (3) |
| + ApoB/ApoA ratio† | 54 | 59 | 60 | 60 | 233 (3) |
| + BMI | 57 | 59 | 60 | 60 | 236 (4) |
| * Basic model: Age, age², fasting time, fasting time², ambient temperature, ambient temperature² and plate ID.  † Not included in the current OLINK Explore panel but measured in other ways among study participants  Abbreviations: FDR=False discovery rate; CVD=Cardiovascular disease; SBP=Systolic blood pressure; BMI=Body mass index. | | | | | |

| eTable 8: Results of phenome-wide association studies for 13 candidate causal proteins | | | | | | | | | |
| --- | --- | --- | --- | --- | --- | --- | --- | --- | --- |
| **Protein name** | **Category** | **Outcome/trait** | **Variant ID** | **Study** | **Ancestry** | **Beta** | **SE** | **p-value** | **n_cases** |
| FURIN | Other traits | Birth weight of first child | chr15:90897856 | Neale B | European | 0.02706 | 0.004588 | 3.68E-09 | — |
| FURIN | Other traits | Illnesses of father: heart disease | chr15:90897856 | Neale B | European | -0.007189 | 0.001253 | 9.71E-09 | 97385 |
| FURIN | Other traits | Illnesses of father: none of the above, group 1 | chr15:90897856 | Neale B | European | 0.007254 | 0.001302 | 2.50E-08 | 109206 |
| FURIN | Other traits | Illnesses of mother: high blood pressure | chr15:90897856 | Neale B | European | -0.007658 | 0.001198 | 1.65E-10 | 94817 |
| FURIN | Other traits | Medication for cholesterol, blood pressure or diabetes: blood pressure medication | chr15:90897856 | Neale B | European | -0.01135 | 0.001607 | 1.65E-12 | 38548 |
| FURIN | Other traits | Medication for cholesterol, blood pressure or diabetes: none of the above | chr15:90897856 | Neale B | European | 0.01127 | 0.001752 | 1.25E-10 | 103004 |
| FURIN | Other traits | Treatment with blood pressure medication | chr15:90897856 | Neale B | European | -0.008728 | 0.001307 | 2.46E-11 | 31488 |
| FURIN | CVD risk factors | Diastolic blood pressure | chr15:90897856 | Neale B | European | -0.02466 | 0.002541 | 2.88E-22 | — |
| FURIN | CVD risk factors | Systolic blood pressure | chr15:90897856 | Neale B | European | -0.02498 | 0.00254 | 8.03E-23 | — |
| FURIN | CVD risk factors | Self-reported hypertension | chr15:90897856 | Neale B | European | -0.01182 | 0.0011 | 6.30E-27 | 87690 |
| FURIN | CVD outcome | Coronary artery disease | chr15:90897856 | Nelson CP | Mixed | -0.05177 | 0.00876 | 3.55E-09 | 10801 |
| FURIN | CVD outcome | Coronary artery disease | chr15:90897856 | van der Harst P | Mixed | -0.0571 | 0.0059 | 4.67E-22 | 122733 |
| FURIN | CVD outcome | Vascular or heart problems diagnosed by doctor: high blood pressure | chr15:90897856 | Neale B | European | -0.01187 | 0.001116 | 1.92E-26 | 91033 |
| FURIN | CVD outcome | Vascular or heart problems diagnosed by doctor: none of the above | chr15:90897856 | Neale B | European | 0.01287 | 0.001146 | 2.75E-29 | 236530 |
| SORT1 | Other traits | Medication for cholesterol, blood pressure or diabetes: cholesterol lowering medication | chr1:109274623 | Neale B | European | 0.01952 | 0.001613 | 1.14E-33 | 35840 |
| SORT1 | Other traits | Medication for cholesterol, blood pressure or diabetes: none of the above | chr1:109274623 | Neale B | European | -0.01295 | 0.001804 | 7.06E-13 | 103004 |
| SORT1 | Other traits | Medication for pain relief, constipation, heartburn: aspirin | chr1:109274623 | Neale B | European | 0.004972 | 0.0008987 | 3.17E-08 | 46946 |
| SORT1 | Other traits | Treatment with atorvastatin | chr1:109274623 | Neale B | European | 0.003661 | 0.0004427 | 1.35E-16 | 10167 |
| SORT1 | Other traits | Treatment with cholesterol lowering medication | chr1:109274623 | Neale B | European | 0.009891 | 0.001178 | 4.58E-17 | 22705 |
| SORT1 | Other traits | Treatment with ezetimibe | chr1:109274623 | Neale B | European | 0.001151 | 0.000199 | 7.32E-09 | 1997 |
| SORT1 | Other traits | Treatment with simvastatin | chr1:109274623 | Neale B | European | 0.00732 | 0.0008197 | 4.27E-19 | 38492 |
| SORT1 | CVD risk factors | Low density lipoprotein | chr1:109274623 | Prins B | European | 0.09 | 0.02 | 1.97E-08 | — |
| SORT1 | CVD risk factors | Low density lipoprotein | chr1:109274623 | GLGC | European | 0.0973 | 0.0057 | 7.04E-59 | — |
| SORT1 | CVD risk factors | Total cholesterol | chr1:109274623 | GLGC | European | 0.07 | 0.0054 | 2.95E-35 | — |
| SORT1 | CVD risk factors | Self-reported high cholesterol | chr1:109274623 | Neale B | European | 0.01183 | 0.0008471 | 2.48E-44 | 41296 |
| SORT1 | CVD outcome | Coronary artery disease | chr1:109274623 | CARDIoGRAM+C4D | Mixed | 0.06849 | 0.0104 | 4.48E-11 | 60801 |
| SORT1 | CVD outcome | Coronary artery disease | chr1:109274623 | van der Harst P | Mixed | 0.0684 | 0.0061 | 3.70E-29 | 122733 |
| SORT1 | CVD outcome | Chronic ischaemic heart disease | chr1:109274623 | Neale B | European | 0.002431 | 0.0004101 | 3.06E-09 | 8755 |
| SORT1 | CVD outcome | Coronary artery disease | chr1:109274623 | Nelson CP | Mixed | 0.06755 | 0.00903 | 7.60E-14 | 10801 |
| PGF | CVD outcome | Coronary artery disease | chr14:75002247 | van der Harst P | Mixed | -0.033 | 0.0056 | 4.88E-09 | 122733 |
| F2R | CVD risk factors | Mean platelet volume | chr5:76750290 | Astle W | European | 0.0253 | 0.003638 | 3.52E-12 | — |
| TFPI | CVD risk factors | Pulse rate | chr2:187467463 | Neale B | European | -0.02187 | 0.00275 | 1.82E-15 | — |
| TFPI | CVD risk factors | Sitting height | chr2:187467463 | Neale B | European | -0.01127 | 0.002064 | 4.75E-08 | — |
| EFNA1 | CVD risk factors | Impedance of leg right | chr1:155133578 | Neale B | European | 0.01236 | 0.002198 | 1.87E-08 | — |
| Abbreviations: SORT1=Sortilin; FURIN=Furin; PGF=Placenta growth factor; TFPI=Tissue factor pathway inhibitor; F2R=Proteinase-activated receptor 1; REG1B=Lithostathine-1-beta; EFNA1=Ephrin-A1; GLGC: The global lipids genetics consortium. | | | | | | | | | |

| eTable 9: Specific phenotypes identified from single-gene KO mouse models for individual proteins | | |
| --- | --- | --- |
| **Protein name** | **Phenotype** | **Category** |
| FURIN | [Cardia bifida](https://identifiers.org/MP:0004187) | [Cardiovascular system phenotype](https://identifiers.org/MP:0005385) |
| FURIN | [Abnormal heart development](https://identifiers.org/MP:0000267) | [Cardiovascular system phenotype](https://identifiers.org/MP:0005385) |
| FURIN | [Failure of heart looping](https://identifiers.org/MP:0004251) | [Cardiovascular system phenotype](https://identifiers.org/MP:0005385) |
| FURIN | [Abnormal cardinal vein morphology](https://identifiers.org/MP:0004783) | [Cardiovascular system phenotype](https://identifiers.org/MP:0005385) |
| FURIN | [Abnormal heart tube morphology](https://identifiers.org/MP:0000270) | [Cardiovascular system phenotype](https://identifiers.org/MP:0005385) |
| TFPI | [Hemorrhage](https://identifiers.org/MP:0001914) | [Cardiovascular system phenotype](https://identifiers.org/MP:0005385) |
| TFPI | [Poor circulation](https://identifiers.org/MP:0001633) | [Cardiovascular system phenotype](https://identifiers.org/MP:0005385) |
| TFPI | [Abnormal thrombosis](https://identifiers.org/MP:0005048) | [Homeostasis/metabolism phenotype](https://identifiers.org/MP:0005376) |
| TFPI | [Abnormal blood coagulation](https://identifiers.org/MP:0002551) | [Homeostasis/metabolism phenotype](https://identifiers.org/MP:0005376) |
| TFPI | [Embryonic lethality during organogenesis, incomplete penetrance](https://identifiers.org/MP:0011108) | [Mortality/aging](https://identifiers.org/MP:0010768) |
| TFPI | [Lethality throughout fetal growth and development, incomplete penetrance](https://identifiers.org/MP:0011109) | [Mortality/aging](https://identifiers.org/MP:0010768) |
| F2R | [Abnormal heart left ventricle morphology](https://identifiers.org/MP:0003921) | [Cardiovascular system phenotype](https://identifiers.org/MP:0005385) |
| F2R | [Abnormal cardiovascular system physiology](https://identifiers.org/MP:0001544) | [Cardiovascular system phenotype](https://identifiers.org/MP:0005385) |
| F2R | [Abnormal sinus venosus morphology](https://identifiers.org/MP:0003228) | [Cardiovascular system phenotype](https://identifiers.org/MP:0005385) |
| F2R | [Hemorrhage](https://identifiers.org/MP:0001914) | [Cardiovascular system phenotype](https://identifiers.org/MP:0005385) |
| F2R | [Abnormal circulating protein level](https://identifiers.org/MP:0005416) | [Homeostasis/metabolism phenotype](https://identifiers.org/MP:0005376) |
| F2R | [Edema](https://identifiers.org/MP:0001785) | [Homeostasis/metabolism phenotype](https://identifiers.org/MP:0005376) |
| F2R | [Increased Tumor Necrosis Factor Receptor Superfamily Member 11b Level](https://identifiers.org/MP:0021032) | [Homeostasis/metabolism phenotype](https://identifiers.org/MP:0005376) |
| F2R | [Embryonic lethality during organogenesis, incomplete penetrance](https://identifiers.org/MP:0011108) | [Mortality/aging](https://identifiers.org/MP:0010768) |
| F2R | [Decreased sensitivity to induced morbidity/mortality](https://identifiers.org/MP:0009764) | [Mortality/aging](https://identifiers.org/MP:0010768) |
| F2R | [Decreased survivor rate](https://identifiers.org/MP:0008770) | [Mortality/aging](https://identifiers.org/MP:0010768) |
| EFNA1 | [Thick aortic valve](https://identifiers.org/MP:0010594) | [Cardiovascular system phenotype](https://identifiers.org/MP:0005385) |
| EFNA1 | [Decreased cardiac muscle contractility](https://identifiers.org/MP:0005140) | [Cardiovascular system phenotype](https://identifiers.org/MP:0005385) |
| EFNA1 | [Abnormal heart left ventricle morphology](https://identifiers.org/MP:0003921) | [Cardiovascular system phenotype](https://identifiers.org/MP:0005385) |
| EFNA1 | [Irregular heartbeat](https://identifiers.org/MP:0001636) | [Cardiovascular system phenotype](https://identifiers.org/MP:0005385) |
| EFNA1 | [Abnormal cardiac outflow tract development](https://identifiers.org/MP:0006126) | [Cardiovascular system phenotype](https://identifiers.org/MP:0005385) |
| PGF | [Abnormal adipose tissue morphology](https://identifiers.org/MP:0000003) | [Adipose tissue phenotype](https://identifiers.org/MP:0005375) |
| PGF | [Decreased total body fat amount](https://identifiers.org/MP:0010025) | [Adipose tissue phenotype](https://identifiers.org/MP:0005375) |
| PGF | [Decreased fat cell size](https://identifiers.org/MP:0009269) | [Adipose tissue phenotype](https://identifiers.org/MP:0005375) |
| PGF | [Decreased gonadal fat pad weight](https://identifiers.org/MP:0009283) | [Adipose tissue phenotype](https://identifiers.org/MP:0005375) |
| ANGPTL1 | [Increased blood urea nitrogen level](https://identifiers.org/MP:0005565) | [Homeostasis/metabolism phenotype](https://identifiers.org/MP:0005376) |
| OBP2B | [Increased circulating cholesterol level](https://identifiers.org/MP:0005178) | [Homeostasis/metabolism phenotype](https://identifiers.org/MP:0005376) |
| REG1B | [Impaired glucose tolerance](https://identifiers.org/MP:0005293) | [Homeostasis/metabolism phenotype](https://identifiers.org/MP:0005376) |
| REG1B | [Increased circulating ldl cholesterol level](https://identifiers.org/MP:0000182) | [Homeostasis/metabolism phenotype](https://identifiers.org/MP:0005376) |
| REG1B | [Abnormal response/metabolism to endogenous compounds](https://identifiers.org/MP:0003638) | [Homeostasis/metabolism phenotype](https://identifiers.org/MP:0005376) |
| REG1B | [Increased circulating hdl cholesterol level](https://identifiers.org/MP:0001556) | [Homeostasis/metabolism phenotype](https://identifiers.org/MP:0005376) |
| REG1B | [Increased circulating cholesterol level](https://identifiers.org/MP:0005178) | [Homeostasis/metabolism phenotype](https://identifiers.org/MP:0005376) |
| REG1B | [Increased sensitivity to induced morbidity/mortality](https://identifiers.org/MP:0009763) | [Mortality/aging](https://identifiers.org/MP:0010768) |
| ASGR1 | [Increased sensitivity to induced morbidity/mortality](https://identifiers.org/MP:0009763) | [Mortality/aging](https://identifiers.org/MP:0010768) |
| ASGR1 | [Increased circulating alkaline phosphatase level](https://identifiers.org/MP:0002968) | [Homeostasis/metabolism phenotype](https://identifiers.org/MP:0005376) |
| ASGR1 | [Decreased bleeding time](https://identifiers.org/MP:0005607) | [Homeostasis/metabolism phenotype](https://identifiers.org/MP:0005376) |
| TNC | [Abnormal serotonin level](https://identifiers.org/MP:0005322) | [Homeostasis/metabolism phenotype](https://identifiers.org/MP:0005376) |
| TNC | [Decreased dopamine level](https://identifiers.org/MP:0005643) | [Homeostasis/metabolism phenotype](https://identifiers.org/MP:0005376) |

| eTable 10: Top 10 KEGG pathways for 361 significantly associated proteins for IHD | | | | |
| --- | --- | --- | --- | --- |
| **Pathways** | **Number of proteins** | **Fold enrichment** | **FDR** | **Proteins involved** |
| Cytokine-cytokine receptor interaction | 53 | 12.453 | 5.24E-40 | CX3CL1 TNFRSF12A TNFRSF1B TNFRSF9 RELT TNFRSF1A THPO CD40 CD40LG TNFSF13B CCL17 IL7 TNFRSF10A TGFB1 CCL7 CCL8 IL1R1 IL1RL1 TNFRSF10B CD70 TNFRSF19 LIF GDF15 EDA2R CCL25 NGF IL6ST IL2RA IL6 CXCL9 ACVRL1 TNFRSF11A XCL1 IL18 CCL28 IL6R CXCL16 TNFSF13 IL24 TGFBR2 CXCL3 CXCL5 CXCL1 IL15 TNFRSF11B CXCL10 CCL13 TNFRSF4 CXCL17 IL1RAP CCL27 TNFSF12 TNFRSF6B |
| PI3K-Akt signalling pathway | 34 | 6.635 | 1.90E-16 | TNC ANGPT2 PDGFB IL7 COMP AREG VWF COL9A1 VEGFA LAMA4 TNR SPP1 FGF23 PGF CDKN1A NGF IL2RA IL6 EGF IGF1R EPHA2 VEGFC BCL2L11 ANGPT1 IL6R ITGA5 TGFA COL6A3 VEGFD EFNA1 F2R PDGFA NTF4 EFNA4 |
| MAPK signalling pathway | 26 | 6.109 | 1.11E-11 | TNFRSF1A ANGPT2 PDGFB PPM1A STK4 TGFB1 AREG VEGFA IL1R1 PLA2G4A FGF23 PGF NGF EGF IGF1R EPHA2 VEGFC ANGPT1 TGFA TGFBR2 VEGFD EFNA1 IL1RAP PDGFA NTF4 EFNA4 |
| Pathways in cancer | 25 | 3.258 | 4.48E-06 | PDGFB STK4 IL7 TGFB1 VEGFA LAMA4 FGF23 PGF CDKN1A IL6ST IL2RA IL6 EGF IGF1R WNT9A VEGFC BCL2L11 IL6R TGFA TGFBR2 IL15 VEGFD F2R MMP1 PDGFA |
| Viral protein interaction with cytokine and cytokine receptors | 24 | 16.746 | 7.69E-21 | CX3CL1 TNFRSF1B TNFRSF1A CCL17 TNFRSF10A CCL7 CCL8 TNFRSF10B CCL25 IL6ST IL2RA IL6 CXCL9 XCL1 IL18 CCL28 IL6R IL24 CXCL3 CXCL5 CXCL1 CXCL10 CCL13 CCL27 |
| RAS signalling pathway | 20 | 5.981 | 6.97E-09 | ANGPT2 PDGFB STK4 VEGFA PLA2G4A FGF23 PGF NGF EGF IGF1R EPHA2 VEGFC ANGPT1 TGFA VEGFD EFNA1 PDGFA LAT NTF4 EFNA4 |
| Focal adhesion | 17 | 5.872 | 1.63E-07 | TNC PDGFB COMP VWF COL9A1 VEGFA LAMA4 TNR SPP1 PGF EGF IGF1R VEGFC ITGA5 COL6A3 VEGFD PDGFA |
| Rap1 signalling pathway | 17 | 5.592 | 3.01E-07 | ANGPT2 PDGFB VEGFA FGF23 PGF NGF EGF IGF1R EPHA2 VEGFC ANGPT1 VEGFD EFNA1 F2R PDGFA LAT EFNA4 |
| Chemokine signalling pathway | 15 | 5.425 | 2.45E-06 | CX3CL1 CCL17 CCL7 CCL8 CCL25 CXCL9 XCL1 CCL28 CXCL16 CXCL3 CXCL5 CXCL1 CXCL10 CCL13 CCL27 |
| Rheumatoid arthritis | 14 | 10.512 | 2.68E-09 | TNFSF13B TGFB1 VEGFA IL6 TNFRSF11A MMP3 IL18 ANGPT1 TNFSF13 CXCL3 CXCL5 CXCL1 IL15 MMP1 |

# eFigure 1: Distribution of concentration levels of 12 proteins showing bimodal distribution pattern


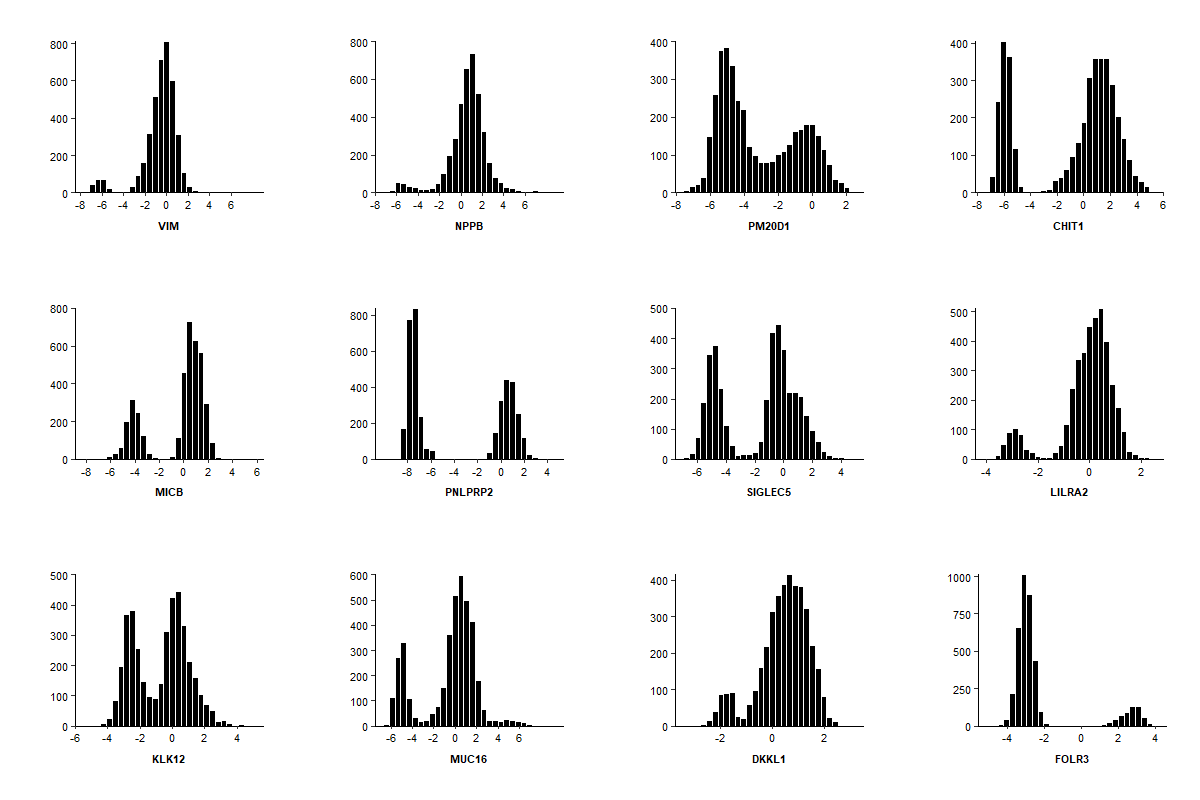


# eFigure 2: Total number and name of the top 10 proteins significantly associated at FDR<0.05 with major CVD risk factors in observational analyses

Model was adjusted for sex, region, age², fasting time, fasting time^2^, ambient temperature, ambient temperature^2^, and plate ID.


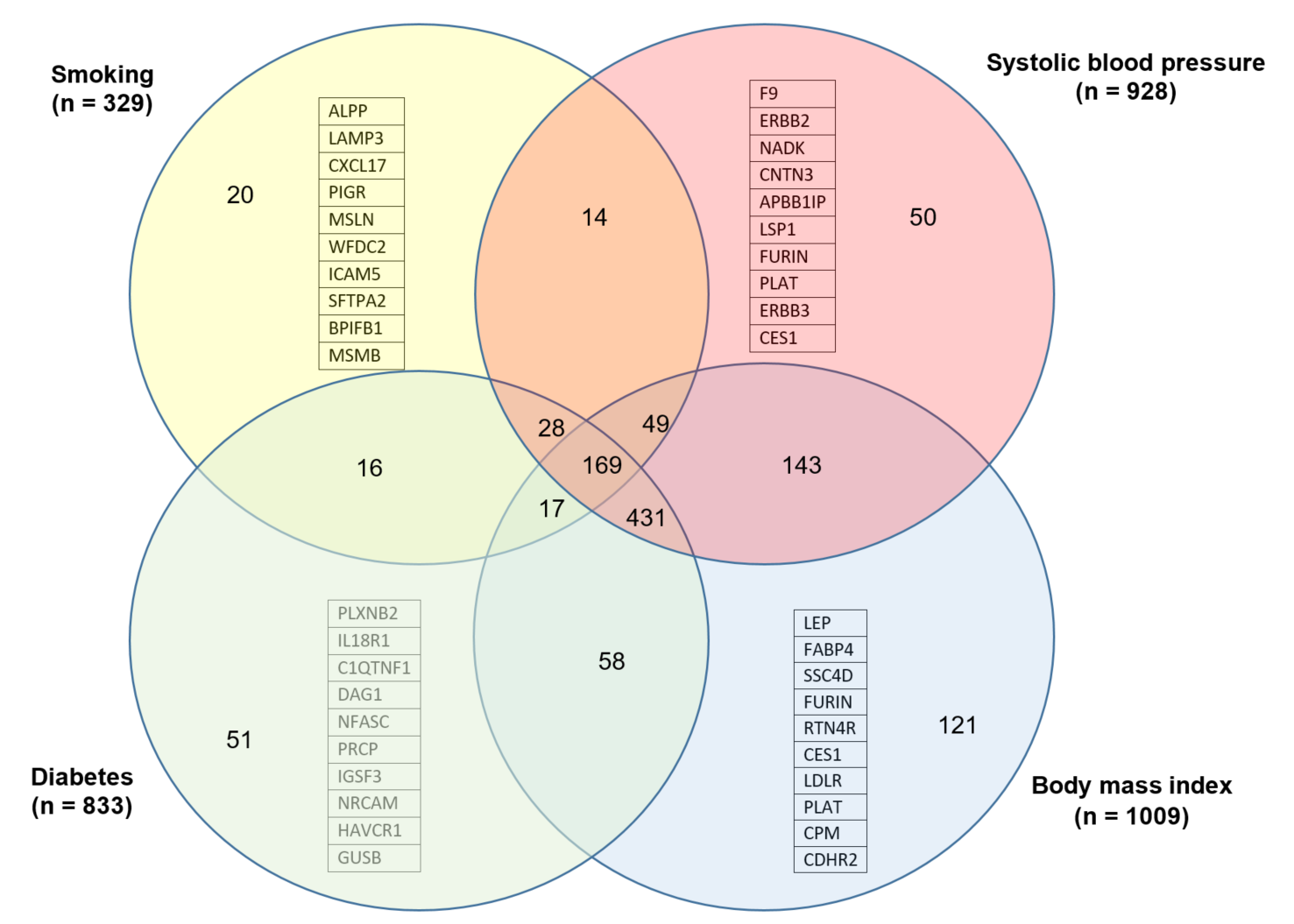


# eFigure 3A: Adjusted HRs for risk of IHD by quartiles of top 25 proteins in observational analyses, in OLINK Cardiometabolic panel

Models were stratified by sex and region, and adjusted for age², fasting time, fasting time^2^, ambient temperature, ambient temperature^2^, plate ID, education, smoking, alcohol consumption, physical activity, SBP, type 2 diabetes, ApoB/ApoA and BMI. The boxes are HRs (indicated by the number above each box) and the vertical lines 95% CIs. The area of the box is inversely proportional to the variance of logHR.


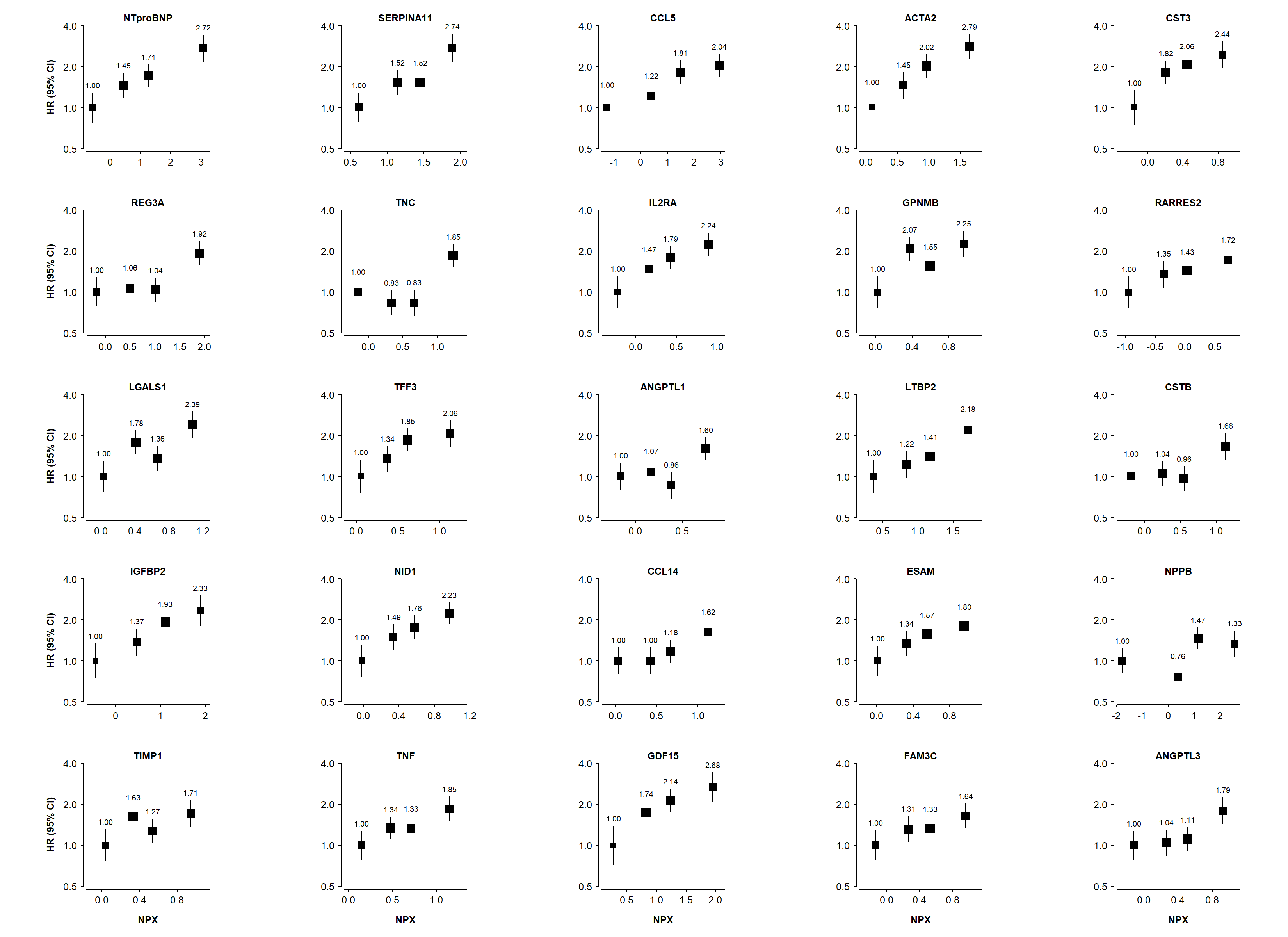


# eFigure 3B: Adjusted HRs for risk of IHD by quartiles of top 25 proteins in observational analyses, in OLINK Inflammation panel

Models were stratified by sex and region, and adjusted for age², fasting time, fasting time^2^, ambient temperature, ambient temperature^2^, plate ID, education, smoking, alcohol consumption, physical activity, SBP, type 2 diabetes, ApoB/ApoA and BMI. The boxes are HRs (indicated by the number above each box) and the vertical lines 95% CIs. The area of the box is inversely proportional to the variance of logHR.


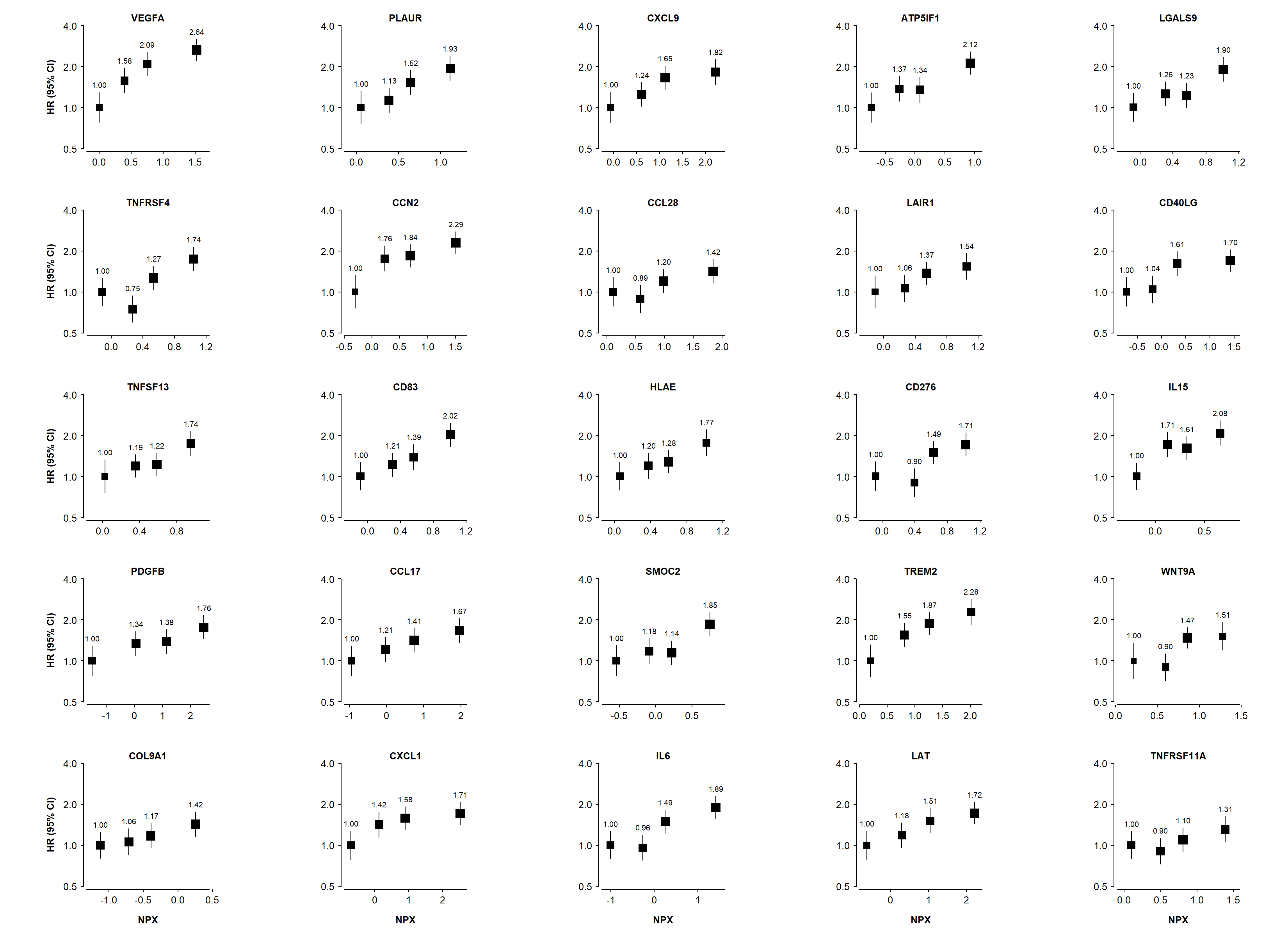


# eFigure 3C: Adjusted HRs for risk of IHD by quartiles of top 25 proteins in observational analyses, in OLINK Neurology panel

Models were stratified by sex and region, and adjusted for age², fasting time, fasting time^2^, ambient temperature, ambient temperature^2^, plate ID, education, smoking, alcohol consumption, physical activity, SBP, type 2 diabetes, ApoB/ApoA and BMI. The boxes are HRs (indicated by the number above each box) and the vertical lines 95% CIs. The area of the box is inversely proportional to the variance of logHR.


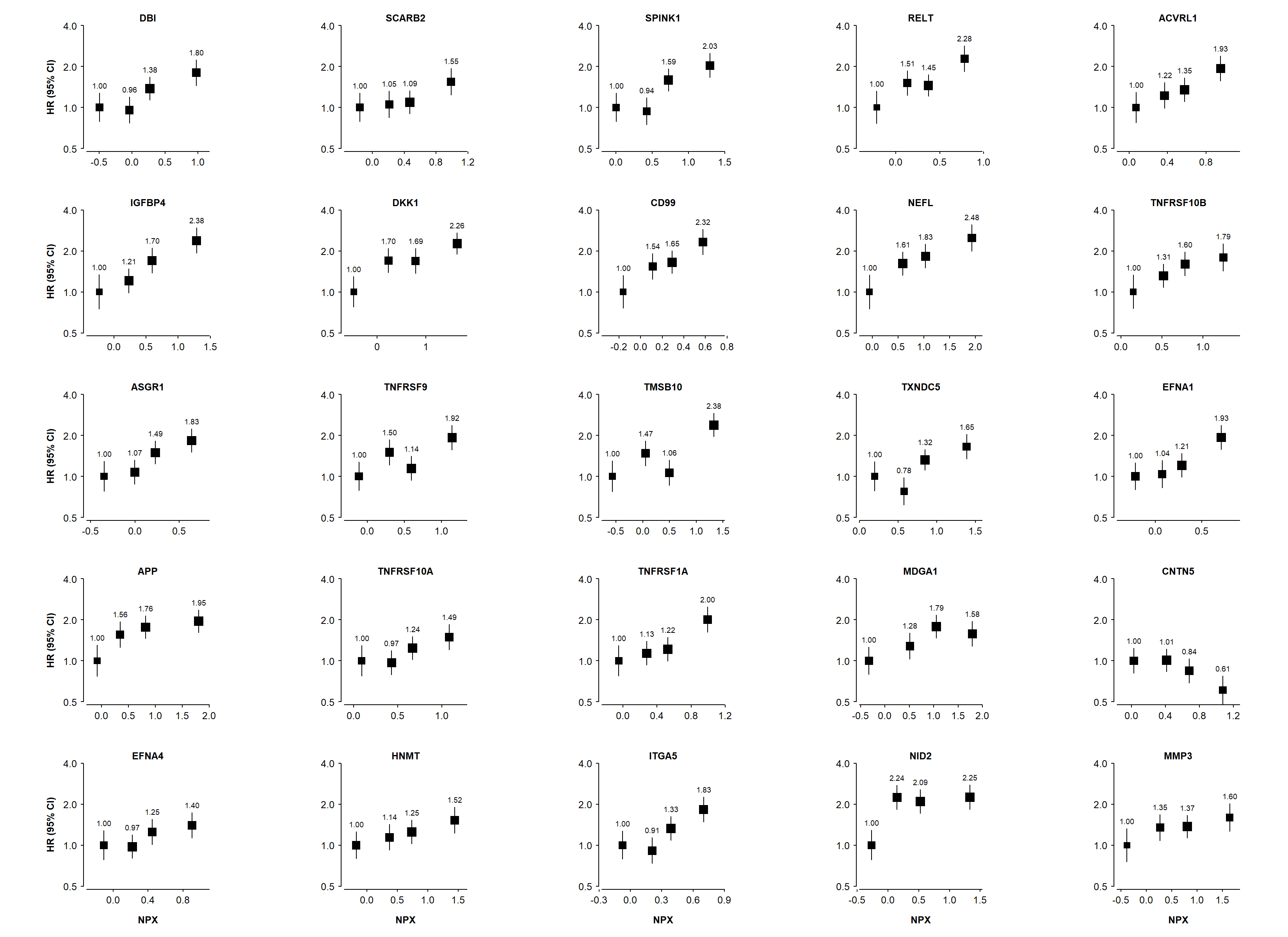


# eFigure 3D: Adjusted HRs for risk of IHD by quartiles of top 25 proteins in observational analyses, in OLINK Oncology panel

Models were stratified by sex and region, and adjusted for age², fasting time, fasting time^2^, ambient temperature, ambient temperature^2^, plate ID, education, smoking, alcohol consumption, physical activity, SBP, type 2 diabetes, ApoB/ApoA and BMI. The boxes are HRs (indicated by the number above each box) and the vertical lines 95% CIs. The area of the box is inversely proportional to the variance of logHR.


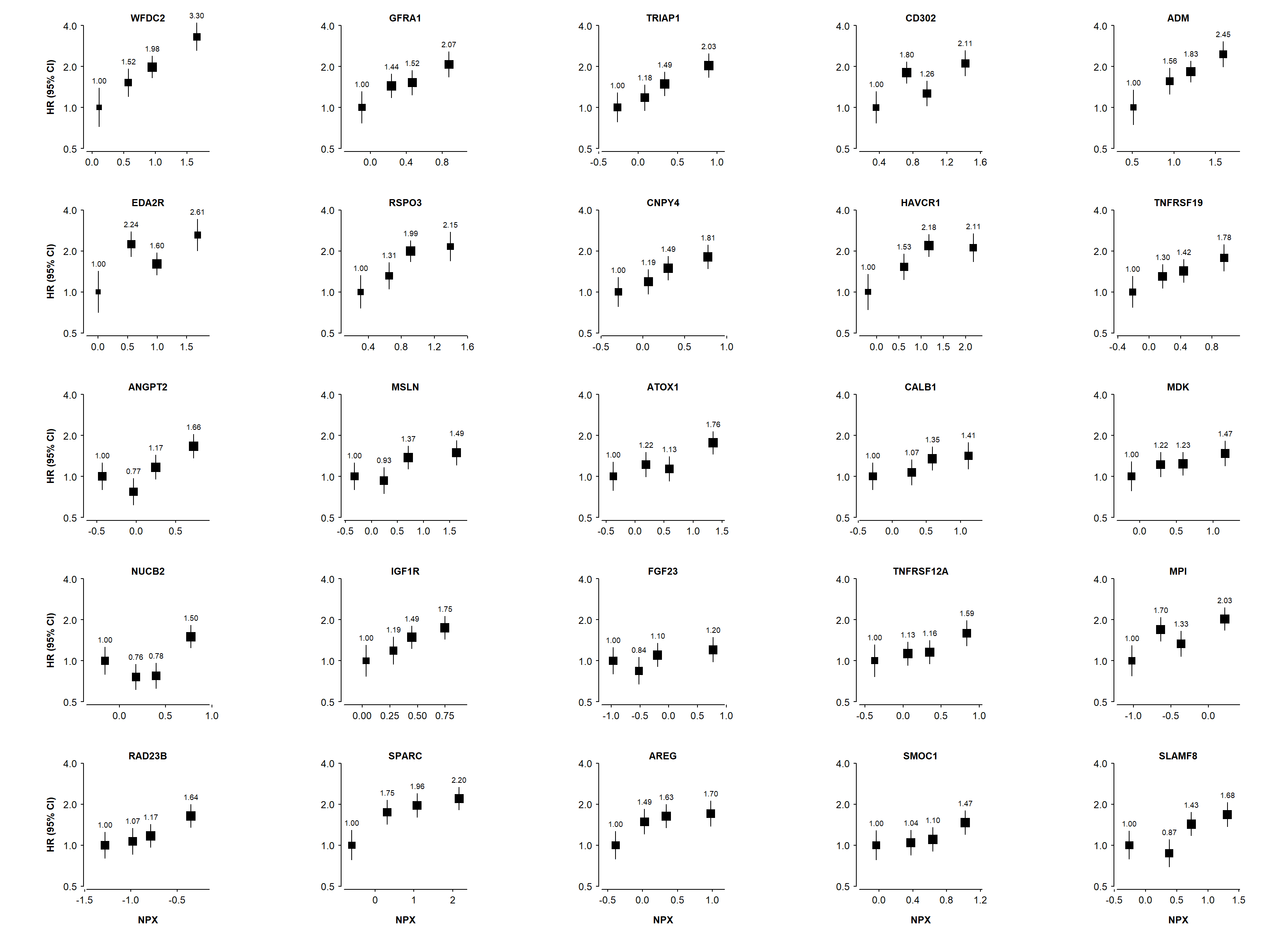


# eFigure 4: Total number of the proteins significantly associated at FDR<0.05 with risk of IHD in case sub-cohort vs. sub-cohort only, in final model of observational analyses

Diagram showing the number of the significant proteins for case sub-cohort vs. sub-cohort only and overlap between them. Models were stratified by sex and region and adjusted for age², fasting time, fasting time^2^, ambient temperature, ambient temperature^2^, plate ID, education, smoking, alcohol consumption, physical activity, SBP, type 2 diabetes, ApoB/ApoA, and BMI.

#
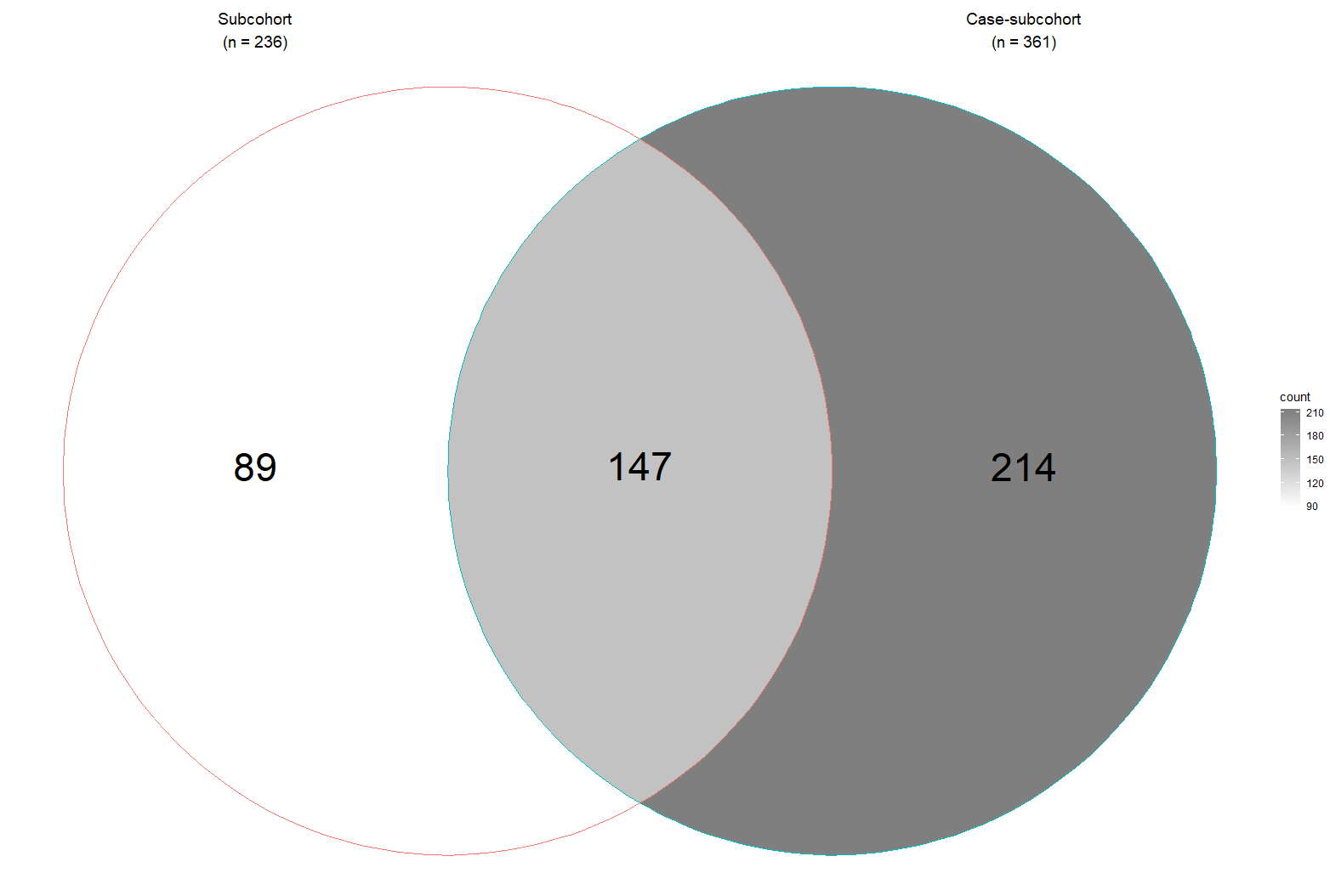


# eFigure 5: Global agreement in effect estimates between observational (CKB) and genetic (CKB & CC4D) analyses

The results in the observational analyses were stratified by sex and region and adjusted for age², fasting time, fasting time^2^, ambient temperature, ambient temperature^2^, plate ID, education, smoking, alcohol consumption, physical activity, SBP, type 2 diabetes, ApoB/ApoA, and BMI. The MR estimates (CKB and CC4D) were based on *Cis*-pQTLs variants identified in CKB for significant protein hits in observational analyses.


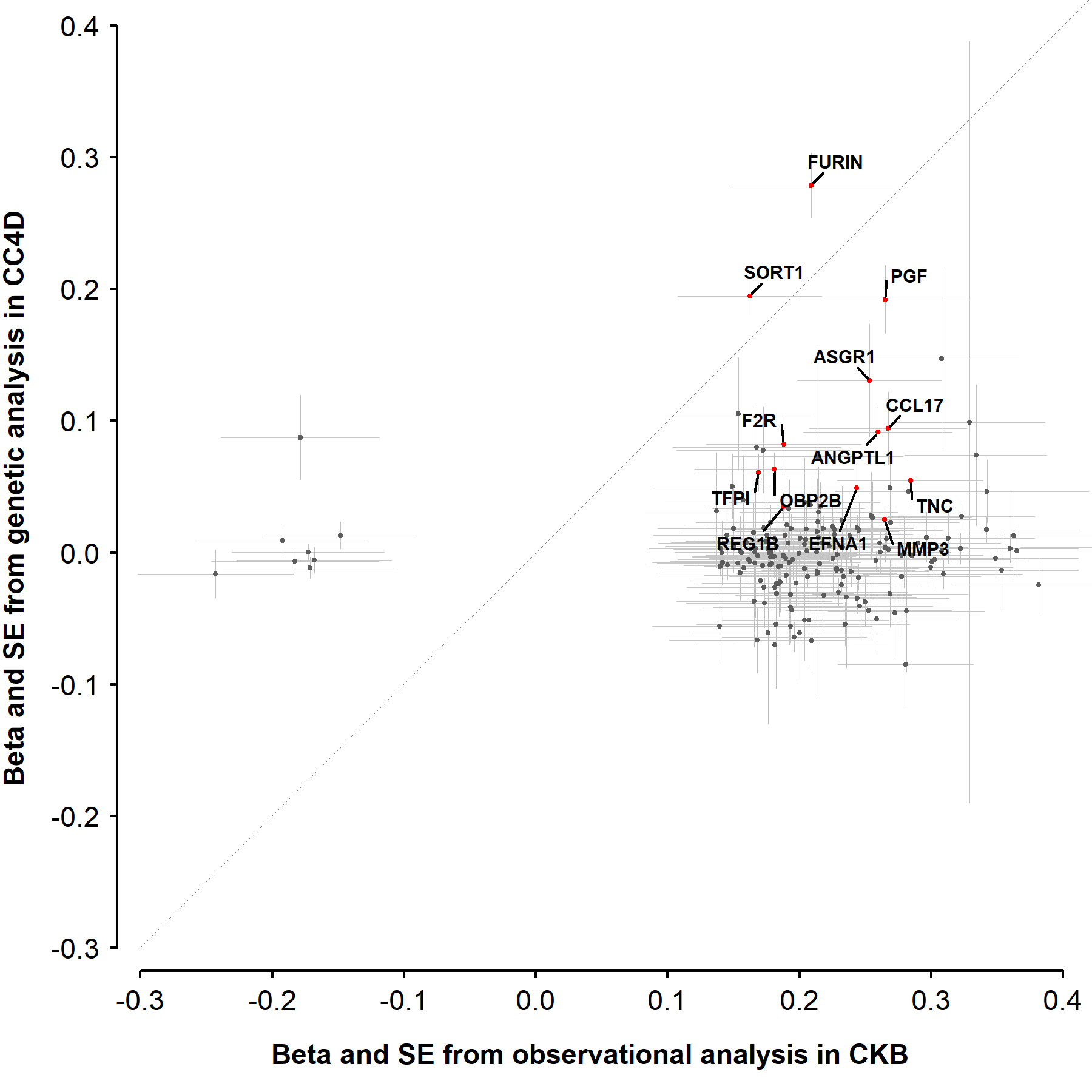


eFigure 6: Adjusted HRs and ORs for risk of IHD associated with 1 SD higher levels of 13 significant proteins in (a) observational (CKB) and (b) genetic (CC4D) analyses, respectively

Observational analyses were stratified by sex and region and adjusted for age², fasting time, fasting time^2^, ambient temperature, ambient temperature^2^, plate ID, education, smoking, alcohol consumption, physical activity, SBP, type 2 diabetes, ApoB/ApoA, and BMI. The boxes are HRs/OR and the horizontal lines are their 95% CIs. The area of the box is inversely proportional to the variance of the log HR.


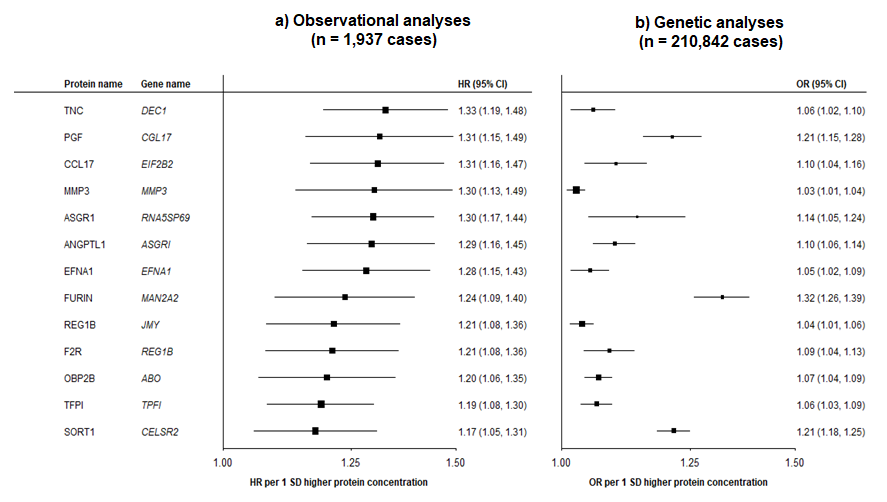


# eFigure 7: Global agreement in effect estimates between observational (CKB) and genetic (CC4D **&** UKB) analyses

The estimates in observational analyses were stratified by sex and region and adjusted for age², fasting time, fasting time^2^, ambient temperature, ambient temperature^2^, plate ID, education, smoking, alcohol consumption, physical activity, SBP, type 2 diabetes, ApoB/ApoA, and BMI. The MR estimates were based on *Cis*-pQTLs variants identified in UKB for protein hits in CKB observational analyses.


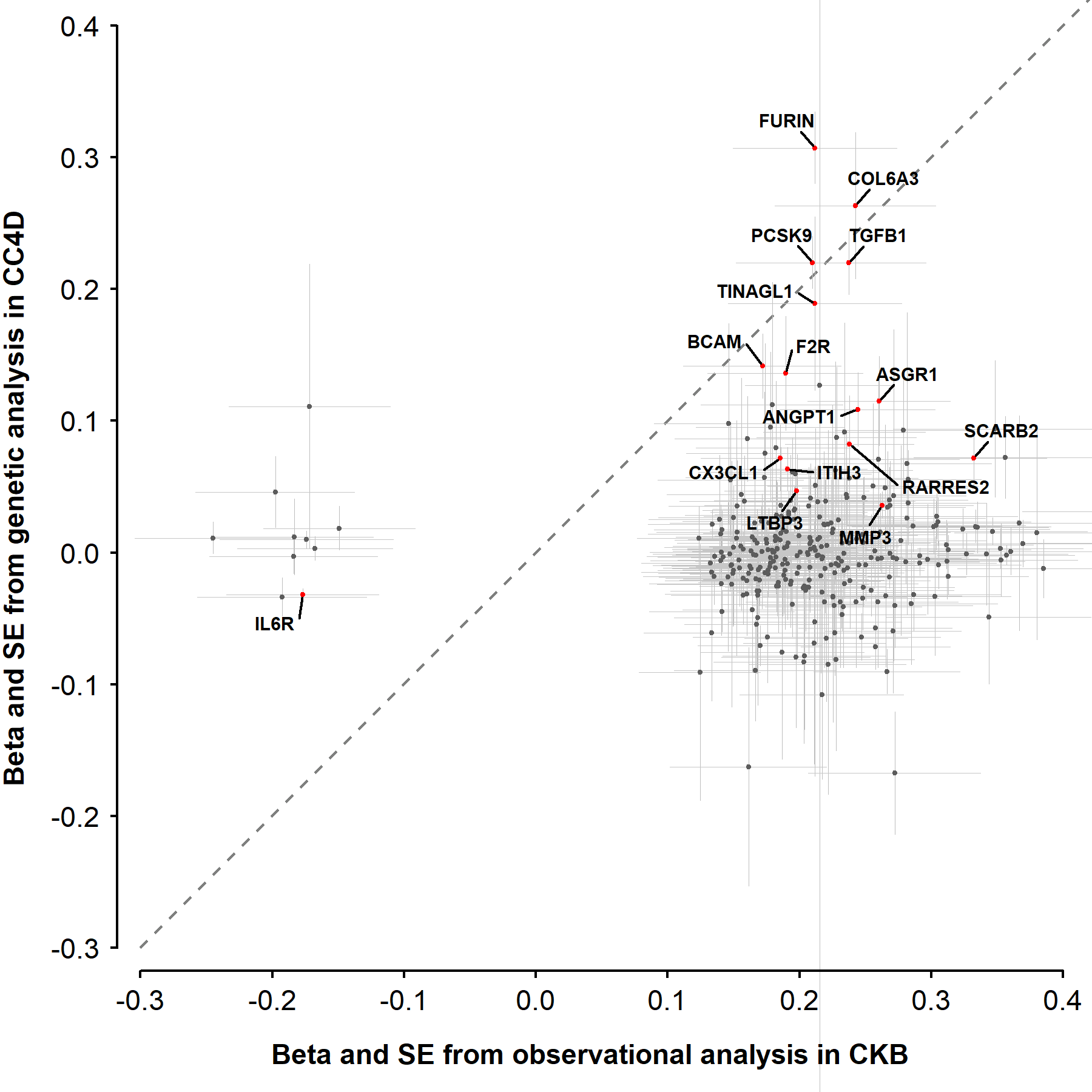


**Refrences:**

1. Lundberg M, Thorsen SB, Assarsson E et al. Multiplexed homogeneous proximity ligation assays for high-throughput protein biomarker research in serological material. Mol Cell Proteomics 2011;10:M110.004978.

2. Prentice RL. A case-cohort design for epidemiologic cohort studies and disease prevention trials. Biometrika 1986;73:1-11.

3. Sun BB, Chiou J, Traylor M et al. Genetic regulation of the human plasma proteome in 54,306 UK Biobank participants. bioRxiv 2022:2022.06.17.496443.

4. Burgess S, Davey Smith G, Davies NM et al. Guidelines for performing Mendelian randomization investigations. Wellcome Open Res 2019;4:186.
